# Tachykinin receptor 3 in the lateral habenula alleviates pain and anxiety comorbidity in mice

**DOI:** 10.3389/fimmu.2023.1049739

**Published:** 2023-01-23

**Authors:** Wen-Wen Zhang, Teng Chen, Shi-Yi Li, Xin-Yue Wang, Wen-Bo Liu, Yu-Quan Wang, Wen-Li Mi, Qi-Liang Mao-Ying, Yan-Qing Wang, Yu-Xia Chu

**Affiliations:** ^1^Department of Integrative Medicine and Neurobiology, School of Basic Medical Sciences, Shanghai Medical College, Institutes of Integrative Medicine, Fudan University, Shanghai, China; ^2^Shanghai Key Laboratory of Acupuncture Mechanism and Acupoint Function, Institute of Acupuncture Research, Fudan University, Shanghai, China; ^3^State Key Laboratory of Medical Neurobiology and MOE Frontiers Center for Brain Science, Institutes of Brain Science, Fudan University, Shanghai, China

**Keywords:** tachykinin receptor 3, lateral habenula, trigeminal neuralgia, anxiety, neurokinin B

## Abstract

The coexistence of chronic pain and anxiety is a common clinical phenomenon. Here, the role of tachykinin receptor 3 (NK3R) in the lateral habenula (LHb) in trigeminal neuralgia and in pain-associated anxiety was systematically investigated. First, electrophysiological recording showed that bilateral LHb neurons are hyperactive in a mouse model of trigeminal neuralgia made by partial transection of the infraorbital nerve (pT-ION). Chemicogenetic activation of bilateral LHb glutamatergic neurons in naive mice induced orofacial allodynia and anxiety-like behaviors, and pharmacological activation of NK3R in the LHb attenuated allodynia and anxiety-like behaviors induced by pT-ION. Electrophysiological recording showed that pharmacological activation of NK3R suppressed the abnormal excitation of LHb neurons. In parallel, pharmacological inhibition of NK3R induced orofacial allodynia and anxiety-like behavior in naive mice. The electrophysiological recording showed that pharmacological inhibition of NK3R activates LHb neurons. Neurokinin B (NKB) is an endogenous high-affinity ligand of NK3R, which binds NK3R and activates it to perform physiological functions, and further neuron projection tracing showed that the front section of the periaqueductal gray (fPAG) projects NKB-positive nerve fibers to the LHb. Optogenetics combined with electrophysiology recordings characterize the functional connections in this fPAG ^NKB^ → LHb pathway. In addition, electrophysiological recording showed that NKB-positive neurons in the fPAG were more active than NKB-negative neurons in pT-ION mice. Finally, inhibition of NKB release from the fPAG reversed the analgesic and anxiolytic effects of LHb *Tacr3* overexpression in pT-ION mice, indicating that fPAG ^NKB^ → LHb regulates orofacial allodynia and pain-induced anxious behaviors. These findings for NK3R suggest the cellular mechanism behind pT-ION in the LHb and suggest that the fPAG ^NKB^ → LHb circuit is involved in pain and anxiety comorbidity. This previously unrecognized pathway might provide a potential approach for relieving the pain and anxiety associated with trigeminal neuralgia by targeting NK3R.

## 1 Introduction

Patients with trigeminal neuralgia are often clinically complicated with emotional disorders such as abnormal anxiety (1). For example, patients with chronic orofacial allodynia typically show resistance and extreme fear when visiting the dentist or drinking cold drinks (2). Pain-related anxiety is caused by pain, and such anxiety can in turn aggravate the pain, thus forming a negative feedback loop. The mechanistic understanding of how these neurological phenomena occur and develop has only begun to emerge in recent years. Thus, it is necessary to explore the cellular, functional, and structural changes at the neural circuit level underlying this neurological comorbidity.

The lateral habenula (LHb) has been identified as a significant nucleus that regulates chronic pain and mood disorders. The LHb regulates negative emotions, and evidence from clinical studies and animal models suggests that the LHb exhibits hyperactivity in major depressive disorder (3, 4). In addition, the LHb is associated with multiple psychiatric morbidities, and the activation of the LHb has been shown to mediate anxiety-like behavior in patients with temporomandibular disorder (5). The LHb also plays a key role in the development of chronic pain in animals and participates in the process of analgesia (6). In a spared nerve injury-induced neuropathic pain model, the LHb receives inputs from the central nucleus of the amygdala and regulates chronic pain (7). Still, efforts should be made to explore how the LHb is involved in the coexistence of chronic pain and related anxiety.

As one of the members of the tachykinin receptor family, tachykinin receptor 3 (NK3R) is encoded by the *Tacr3* gene and belongs to family of typical G-protein–coupled receptors (8). Most of the previous studies on NK3R showed that it has an important role in regulating reproductive functions through its activities at the hypothalamic, pituitary, and gonadal levels (9). However, whether NK3R affects other neurological disorders has been less studied. Anatomic evidence suggests that NK3R is widely expressed in the central nervous system and is involved in the development of neurological diseases including chronic pain, mood disorders, neurodegenerative diseases, and so on (10, 11). Compared to the other tachykinins, NK3R is preferentially activated by its high-affinity ligand neurokinin B (NKB), which belongs to the tachykinin family, the most intensively studied members of which include substance P, neurokinin A, and NKB (12). Extensive studies have reported that substance P of the tachykinin family is released from primary sensory afferent terminals as an excitatory neurotransmitter and that it binds to NK1R to mediate nociceptive transmission (13). However, whether and how NKB and NK3R are associated with nociceptive transmission has been less studied. NK3R has been shown to have significant anti-anxiety effects in rodents, including mice and rats (14). Activating NK3R by the intracerebroventricular agonist senktide has anxiolytic effects, and inhibition of NK3R induces anxiety-like behaviors (15, 16). In addition, NK3R is also involved in anxiety in different brain regions, such as the amygdala, dorsal periaqueductal gray, and hippocampus (10, 17, 18). However, whether and how NK3R in the LHb is related to pain and comorbid anxiety remains unknown.

Here, chemogenetics, pharmacology, electrophysiology, calcium imaging, western blotting, neuron projection tracing, and behavior tests were used to elucidate whether and how NK3R in the LHb mediates chronic pain and the accompanying anxiety. We show that LHb neurons were activated by inhibition of NK3R and that abnormal excitation of LHb neurons in mice with pT-ION of the trigeminal nerve was suppressed by activating NK3R. Based on the “abnormal excitation or not” of LHb neurons, NK3R was shown to regulate chronic pain and anxiety in a trigeminal neuralgia model. We further found NKB^+^ projections from the front section of the periaqueductal gray (fPAG) to the LHb, and these projections are significant for the regulation of orofacial allodynia and comorbid anxiety by NK3R in the LHb.

## 2 Methods

### 2.1 Animals and the chronic orofacial pain model

Adult male C57BL/6j mice at 6–8 weeks of age and weighing 20–25 g was purchased from the Experimental Animal Center of the Chinese Academy of Sciences (Shanghai, China). The reason we used male mice is because our previous work on trigeminal neuralgia was all done in male mice. Tac2-Cre mice were a gift from Prof. Tian-Wen Huang at the University of Chinese Academy of Sciences with the permission of Prof. Qiu-Fu Ma at Harvard University and were generated by crossing Tac2-Cre males with Tac2-Cre females. For Tac2-Cre mice, both sexes were used for all experiments due to the slow reproduction rate, and the data for the two sexes were then combined. The animals were randomly divided into different groups with 6 animals in each cage. Before each experimental manipulation, the mice were acclimated to the controlled environmental conditions for 1 week (with temperature 22 ± 1°C, humidity 50%–60%, light-dark cycle at 12:12 h), and mice had access to food and water ad libitum. All experimental manipulations were in accordance with the requirements of the Animal Care and Use Committee of Fudan University and the International Association for the Study of Pain, and we sought to minimize the suffering of animals as much as possible. The sample size of each experimental group was determined by our previous work, and the key experiments were repeated with the calculated sample size.

For the pT-ION model, mice were deeply anesthetized with sodium pentobarbital (50 mg/kg, i.p.) while lying in a supine position on a soft surgical mat. The oral cavity of the mice was fully exposed, and pT-ION was performed through the left intraoral approach. Starting from the first molar, a 3–5 mm incision was made in the palatal buccal mucosa with a sterile surgical blade. Submucosal tissue was separated by microforceps, and the infraorbital nerve was gently separated with a bent glass needle. A 2–3 mm section of the deep branch of the infraorbital nerve was cut and removed, and the incision was closed and sutured. The animals were allowed to recover from anesthesia on a warm, soft cushion. The sham-operated animals were only subjected to nerve exposure. Animals in poor health were excluded by comprehensively measuring their body weight, mental state, and exercise ability prior to the operation. Sterile practices were strictly followed during the operation, and the animals were closely observed for postoperative infection or other complications.

### 2.2 Behavioral tests of allodynia

The behavioral tests were double-blinded, meaning that group assignments and trial tests were performed by different experimenters. Before all behavioral tests, animals were acclimated to the test room environment (quiet, temperature 22 ± 1°C, humidity 50%–60%, soft lighting) 2 days in advance, and all tests were conducted between 2 p.m. and 5 p.m. For pain-related behavior, mice were acquainted with the researchers 2 days in advance, and the acetone test was performed at least 30 minutes after the von Frey test.

For the orofacial allodynia test, the mice were gently habituated to the experimenter’s hand. A series of von Frey filaments (0.02 g, 0.04 g, 0.07 g, 0.16 g, 0.4 g, 0.6 g, 1.0 g, and 1.4 g; Stoelting, USA) were applied to the facial V2 and V3 skin. Starting from the threshold of 0.16 g, each von Frey filament was applied once every 5 seconds for a total of five times. Each application time was 3 seconds, and the hair filament was bent into a semicircle for effective application. An aversive response from the animal was considered a positive response. Filaments with three positive responses out of five stimuli were defined as the pain threshold. For mechanical testing in the paw area, animals were acclimated to the test environment 2 days in advance, and the mice were placed on a metal mesh floor covered with clear glass, and the experiment was carried out while the mice were quiet. A series of von Frey fibers (0.02 g, 0.04 g, 0.07 g, 0.16 g, 0.4 g, 0.6 g, 1.0 g, 1.4 g) were pressed on the mid-planar surface of the hind paw, and the fibers were bent into a semicircle five times every 3 seconds. The filaments that caused the mouse foot retraction reflex were recorded as the positive threshold, and the interval between each application was 5 seconds. The 50% withdraw threshold (g) was evaluated as follows: maximum bending force (BF) value − [(maximum BF value − minimum BF value)/(positive rate of the maximum BF − positive rate of the minimum BF)] * (positive rate of the maximum BF − 50%).

For the orofacial cold allodynia test, the mice were pre-acclimated in a clear black glass box (8 cm × 8 cm × 10 cm) for 40 minutes per day for 2 days. A total of 50 μl of acetone was lightly sprayed onto the skin of the affected side in the V3 area *via* a microsyringe (Hamilton, Reno, NV). The total orofacial wiping time was recorded for 2 minutes. Animals with cold allodynia had an increased wiping time after exposure to acetone compared with sham-operated animals. For the paw cold allodynia test, the mice were placed on a wire net floor with a plexiglass chamber cover. The same as for the cold allodynia test in the V3 area, a 50 μl drop of acetone was applied to the mid-plantar surface of the hind paw. The total paw licking or lifting time was obtained within a cut off time of 1 min. Mice with cold allodynia presented increased licking or lifting time after exposure to acetone compared to control group and baseline values.

### 2.3 Behavioral tests of anxiety-like behavior

Anxiety-like behaviors were tested in a double-blinded manner. For anxiety-like behaviors, mice were acclimated in the room where the test bed was located 2 days in advance. Once an animal entered the platform, a video tracking system (Shanghai Mobile Data Information Technology, Shanghai, China) recorded the animal’s behavior in real time for subsequent analysis.

For the elevated plus maze (EPM), mice were placed in the center platform (6 cm × 6 cm) of the maze consisting of four arms (6 cm × 30 cm), including two closed arms (20 cm) and two open arms. The maze was at 40 cm above the ground. The animals were allowed to roam freely in the maze for 5 minutes. The percentages of open arm time (time in open arms/time in total arms × 100%), open-arm entries (open entries/total entries × 100%), and open-arm distance (open distance/total distance × 100%) were calculated to assess how anxious the animals were.

For the open field test (OFT), the animals were allowed to explore freely in an open box (50 cm × 50 cm × 40 cm) for 5 minutes. The time the mice spent in the central square (25 cm × 25 cm), the central distance, and the total distance were recorded and used to assess anxiety. After each animal was tested, the area was cleaned with 75% ethanol to remove olfactory cues from the instrument, and the ethanol was allowed to fully volatilize before the next animal was tested.

### 2.4 Virus injection, chemogenetics, and optogenetics

The mice were anesthetized with sodium pentobarbital (50 mg/kg, i.p.) before fixation and were placed in a stereotactic device after the righting reflex disappeared, and a total of 150–200nl viral particles (~10^12^ infectious units per ml) were injected into the target nuclei through a microsyringe (2.5 µl, Hamilton Company, USA) using an infusion pump (Stoelting, USA) at a rate of 50 nl per min after drilling a hole into the skull surface. The specifics of the viral particles and their uses are described below.

For chemogenetics, rAAV-CaMKIIa-hM3D(Gq)-mCherry-WPREs-pA (AAV2/9, >2 × 10^12^ vg/ml, 150 nl) was bilaterally delivered into the LHb at bregma (−1.75 mm), midline (± 0.45mm), and the skull surface (−2.85 mm) and rAAV-CaMKIIa-DIO-hM4D(Gi)-mCherry-WPRE-hGH (AAV2/9, 5 × 10^12^ vg/ml, 150 nl) was bilaterally delivered into the fPAG at bregma (−2.70 mm), midline ( ± 0.25 mm), and the skull surface (−3.75 mm). Three weeks after the injection of virus, clozapine-N-oxide (CNO, 2.5 mg/kg, i.p.) was administered 45–55 minutes prior to the test. For optogenetics, pAAV-EF1a-DIO-hChR2(H134R)-mCherry-WPRE (AAV2/9, 5 × 10^12^ vg/ml, 150 nl) was bilaterally delivered into the fPAG at bregma (−2.70 mm), midline ( ± 0.25 mm), and the skull surface (−3.75 mm). Three weeks after the injection of virus, 473 nm light stimulation was administered to the LHb during electrophysiological recording.

For overexpression of Tacr3 in the LHb, pAAV-CMV-EGFP-2A-Tacr3-3FLAG (1 × 10^12^ vg/ml, 200 nl) was bilaterally delivered into the LHb. Mice were allowed 3 weeks for transgene expression and recovery.

For neuron projection tracing, the Cre-dependent retro-rAAV-EF1a-DIO–EGFP (AAV2/9, 5.48 × 10^12^ vg/ml, 150 nl) was bilaterally injected into the LHb of the Tac2-Cre mice to look for upstream sources of NKB in the LHb. The anterograde Cre-dependent virus rAAV-EF1a-DIO–mCherry-hGH (AAV2/9, >2 × 10^12^ vg/ml, 150 nl) was bilaterally injected into the fPAG of the Tac2-Cre mice to verify that fPAG does indeed project NKB+ fibers to the LHb. For Cre-dependent anterograde trans-monosynaptic tracing, rAAV-hSyn-CRE-EGFP-WPRE-hGH (AAV2/1, 1.12 × 10^13^ vg/ml, 150 nl) was bilaterally injected into the fPAG and rAAV-EF1α-DIO-EGFP-WPRE-hGH (AAV2/9, 5.66 × 10^12^ vg/ml, 150 nl) was bilaterally injected into the LHb of wild-type (WT) mice to further confirm the fPAG–LHb projection. To determine what kind of neurons the NKB+ projections from the fPAG to the LHb have synaptic connections with, rAAV- EF1α-DIO-FLP-WPRE-hGH (AAV2/1, 1.28 × 10^13^ vg/ml, 150 nl) was bilaterally injected into the fPAG and rAAV-EF1α-fDIO-EGFP-WPRE-hGH (AAV2/9, 5.31 × 10^12^ vg/ml, 150 nl) was bilaterally injected into the LHb of the Tac2-Cre mice. All of the viruses were expressed in the animals’ brains for 3 weeks before sampling.

### 2.5 Cannula implantation and intracerebral drug infusion

Mice were anesthetized with sodium pentobarbital (50 mg/kg, i.p.) and placed in a stereotaxic apparatus. The stainless-steel cannula guide with double parallel pipeline (RWD Life Science, Shenzhen, China) consisted of an outer tube (O.D. 0.41 mm, I.D. 0.25 mm/C.C0.8/B7.8/M3.5) and an inner tube (O.D. 0.21 mm, I.D. 0.11 mm/C.C0.8/G1 = 0.5 mm). The outer tube was bilaterally embedded into the LHb at bregma (−1.75 mm), midline ( ± 0.45 mm), and the skull surface (−2.35 mm) and was secured to the skull surface by dental cement. Mice were allowed to recover for a week after implantation to ensure that the trauma to the mice had been repaired. The inner tube was embedded into the LHb at bregma (−1.75 mm), midline ( ± 0.45mm), and the skull surface (−2.85 mm) prior to the behavior tests by inserting it into the outer tube. Mice were anesthetized with isoflurane, and a microsyringe (2.5 µl, Hamilton Company, USA) pre-filled with the injection solution was mounted to connect the injection cannula with an infusion pump (Stoelting, USA) set at a delivery rate of 50 nl/min (250 nl total volume). After the drug injection, the animal was placed in a cage for wakefulness recovery, and the behavioral test was performed 5–10 minutes after the animal was awake. On day 21 after pT-ION was established, the effects of the injected drugs on orofacial allodynia were determined. Two days were allowed for the drug to metabolize. On day 23 and day 25 after pT-ION, the EPM and OFT were performed on the same group of mice to test the effects of drugs on anxiety-like behaviors. The following drugs were administered: SB-222200 (250 mM), senktide (250 mM), and vehicle (10% DMSO, 40% PEG300, 5% Tween-80 + 45% saline).

### 2.6 Immunofluorescence

Mice were deeply anesthetized with sodium pentobarbital (100 mg/kg, i.p.), the chest was opened, and saline and 4% paraformaldehyde were perfused into the systemic circulation through the ascending aorta. Mouse brains were dissected intact, post-fixed with 4% paraformaldehyde overnight at 4°C, and then transferred to a 30% sucrose solution for 1–2 weeks. The brain was cut into 30–40 μm slices using a freezing microtome (Leica 2000, Germany). For spontaneous fluorescence, the slices were washed three times with 0.3% Triton X-100, 15 minutes at a time, and then cover-slipped. The nuclei were stained with 4’,6-diamidino-2-phenylindole (DAPI) Fluoromount-G^®^ (0100–20, Southern Biotech, Birmingham, AL, USA). For co-localized staining, the slices were blocked for 2 h at room temperature and were then incubated for 12 h at 4°C with rabbit anti-glutamate (1:500 dilution, Sigma) and rabbit anti-GABA (1:500 dilution, Sigma). After washing away unbound primary antibodies, the brain slices were incubated with the corresponding Alexa Fluor 594-conjugated secondary antibody for 2 hours (1:1000 dilution; Invitrogen). The brain slices were mounted on slides and then sealed and stained with DAPI. Images were acquired on a multiphoton laser point scanning confocal microscopy system (FV1000, Olympus, Tokyo, Japan).

### 2.7 Western blot

After cardiac perfusion with ice-cold PBS, the bilateral LHb was collected and proteins were freshly extracted with buffer containing 1% phenylmethylsulfonyl fluoride and a protease/phosphatase inhibitor cocktail (CST, USA). The protein suspension was collected after hypercentrifugation (12,000 × rpm, 20 min), and the concentration was determined using the bicinchoninic acid protein assay (Thermo Scientific, USA). The protein samples were stored in the protein loading buffer for the next experiment. Total proteins were separated according to their molecular weight using 10% sodium dodecyl sulfate-polyacrylamide gel electrophoresis. The isolated proteins were transferred to polyvinylidene fluoride membranes that were blocked in 5% nonfat dry milk for one hour at room temperature. The membranes were incubated with the following primary antibodies overnight at 4°C: rabbit p-CaMKII (1:1000 dilution, CST), mouse anti-p-CamKII (1:1000 dilution, CST), rabbit anti-PKA C-α (1:1000 dilution, CST), rabbit anti-p-PKA (1:1000 dilution, CST), rabbit anti-NK3R (1:500 dilution, NOVUS), rabbit anti-GAPDH (1:10,000 dilution, Proteintech), and rabbit anti-β-tubulin (1:5000 dilution, CST). After washing, the polyvinylidene fluoride membranes were incubated with specific horseradish peroxidase-conjugated secondary antibodies (1:10000 dilution, Proteintech) for 1 h at room temperature. Protein images were acquired on an ImageQuant LAS4000 mini image analyzer (GE Healthcare, UK). The Quantity One Analysis Software (Version 4.6.2, Bio-Rad Laboratories, Hercules, USA) was used to analyze the intensities of the protein bands, and statistical analysis was performed.

### 2.8 Electrophysiology

For brain slice preparation, the NMDG artificial cerebrospinal fluid consisted of 93 mM N-methyl-d-glucamine (NMDG), 20 mM HEPES, 3 mM Na-pyruvate, 10 mM MgSO4, 30 mM NaHCO3, 25 mM glucose, 2.5 mM KCl,2 mM thiourea, 5 mM Na-ascorbate, 1.2 mM NaH2PO4, 0.5 mM CaCl2, and 3 mM glutathione. The pH of the NMDG artificial cerebrospinal fluid was adjusted to 7.3–7.4 with HCl and KOH, and the osmotic pressure was maintained at 300–305 mOsm/kg. The formula of HEPES artificial cerebrospinal fluid was 2 mM thiourea, 2.5 mM KCl, 92 mM NaCl, 30 mM NaHCO3, 1.2 mM NaH2PO4, 25 mM glucose, 20 mM HEPES, 5 mM Na-ascorbate, 2 mM CaCl2, 3 mM Na-pyruvate, 3 mM glutathione, and 2 mM MgSO4 (osmolarity 300–305 mOsm/kg, pH7.3–7.4). Perfusion artificial cerebrospinal fluid consisted of 2.4 mM CaCl2, 3 mM KCl, 20 mM NaHCO3, 1.3 mM MgSO4, 1.2 mM KH2PO4, 129 mM NaCl, and 10 mM glucose (osmolarity 300–305 mOsm/kg, pH7.3–7.4). Mice were anesthetized with sodium pentobarbital (50 mg/kg, i.p.), and 30 ml of ice-cold oxygenated modified NMDG artificial cerebrospinal fluid was perfused into the systemic circulation through the left apex of the heart. Mouse brains were dissected and incubated in ice-cold oxygenated modified NMDG artificial cerebrospinal fluid. Brain coronal sections containing the LHb or fPAG were sectioned on a vibrating microknife (VT1200, Leica) at 0.2 mm/s, and each brain slice was 300 μm thick. The brain slices were initially incubated in NMDG artificial cerebrospinal fluid for 15 min at 33°C, followed by HEPES artificial cerebrospinal fluid for at least 1 h at 25°C. Brain sections were transferred to a circular chamber and continuously perfused with standard artificial cerebrospinal fluid at 32°C, and the perfusion rate was maintained at 3–4 ml/min followed by whole-cell patch-clamp recordings. The experimental operation was double blinded.

For the electrophysiological recording, an infrared differential-interference contrast microscope (BX51WI, Olympus) equipped with a 40× water immersion objective was used to observe neurons in brain slices, and neurons in the LHb were imaged using an infrared camera connected to the video monitor. A four-stage horizontal puller (P1000, Sutter Instruments) was used to prepare a glass microelectrode with 3–5 MΩ resistance. A Multiclamp 700B amplifier collected the neuronal electrical signals, and the signals were analyzed using Clampfit 10.7 software (Molecular Devices). Current-evoked firing (minimum current injections at –10pA, 11–12 current steps, every step at 10 pA, and a duration of 300 ms), spontaneous firing and the resting membrane potential were recorded in current-clamp mode (I = 0 pA), and for the synaptic transmission recording neurons were held at −70 mV using the voltage clamp mode. The data were collected from the neurons with the appropriate input resistance (more than 500 MΩ) and series resistance (less than 50 MΩ). The rheobase of the action potential was defined as the minimum current to elicit an action potential. The intracellular solution was prepared in advance and contained 10 mM HEPES, 0.5 mM EGTA, 15 mM KCL, 125 mM K-gluconate, 2 mM Mg-ATP, 10 mM Na2-phosphocreatine, and 0.5 mM Na-GTP (osmolarity 285-290 mOsm/kg, pH7.2) and then added to the clamp pipettes. SB-222200 (250 mM) and senktide (250 mM) were added to the perfusion artificial cerebrospinal fluid to inhibit or activate NK3R, respectively.

For optogenetically evoked EPSC recording, optical stimulation was performed using a laser (Shanghai Fiblaser Technology Co., Ltd. China) through an optical fiber (diameter, 200 µm, Newdoon, Hangzhou) positioned 0.3 mm above the surface of the target brain region. LHb neurons were held at −70 mV using the voltage clamp mode, and blue light stimulation (473 nm, 60 µW, and 20 Hz continuous stimulation for 1–2 s for the activation of synapses projecting from fPAG NKB-positive neurons) was administered to the LHb after the postsynaptic current firing was stable.

### 2.9 Primary cultures of hippocampal neurons

Primary hippocampal neurons were extracted from 18-day-old C57BL/6J mouse embryos. The brains of the embryonic mice were incubated in ice-cold Hanks’ balanced salt solution (HBSS, Life Technologies, USA). Hippocampi were separated from other brain tissues and dissociated and triturated in 0.15% trypsin for 20–30 min at 37°C. Cells were transferred (7 × 10^5^ cells/cm^2^) to a 6-well plate coated with poly-D-lysine (Sigma, USA) and incubated at 37°C in 5% CO2 and 90% humidity with neurobasal medium containing B27 supplement (ThermoFisher, USA), 100 μg/ml streptomycin (Sigma, USA), GlutaMAX (Gibco, UK), 100 U/ml penicillin (Sigma, USA), and 10% heat-inactivated FBS (Sigma, USA). Half of the neuronal medium was replaced every other day.

### 2.10 Live-cell Ca^2+^ imaging

Well-grown hippocampal neurons were preincubated with 4 μM Fura-2 AM (Life Technologies) for 1 h. Fura-2-loaded cells were rinsed with HBSS prior to formal imaging. A Nikon Ti-E microscope with NIS-elements imaging Software (Nikon, USA) was used to record fluorescence under excitation at 340 nm and 380 nm wavelengths. The fluorescence intensity (Fura-2 ratios, F340/F380) reflects the reaction intensity of the cell.

### 2.11 Statistical analysis

All group data are shown as the mean ± standard error of the mean (SEM). For the anxiety-like behavior test, electrophysiological results, Ca^2+^ imaging experiments, western blotting results, and other comparisons between two groups, unpaired Student’s t-tests were used to analyze statistical differences. For the von Frey and acetone tests, two-way repeated-measures ANOVA followed by Tukey’s multiple comparison test was used to analyze differences between groups. Multiple groups were compared using one-way ANOVA followed by Dunnett’s *post hoc* multiple comparison test. A p-value < 0.05 was considered as the threshold of significance in all tests. GraphPad Prism 11 (San Diego, USA) was used for the statistical analyses.

## 3 Results

### 3.1 Orofacial allodynia and anxiety-like behaviors were associated with abnormal excitation of the LHb

**Figure 1:**
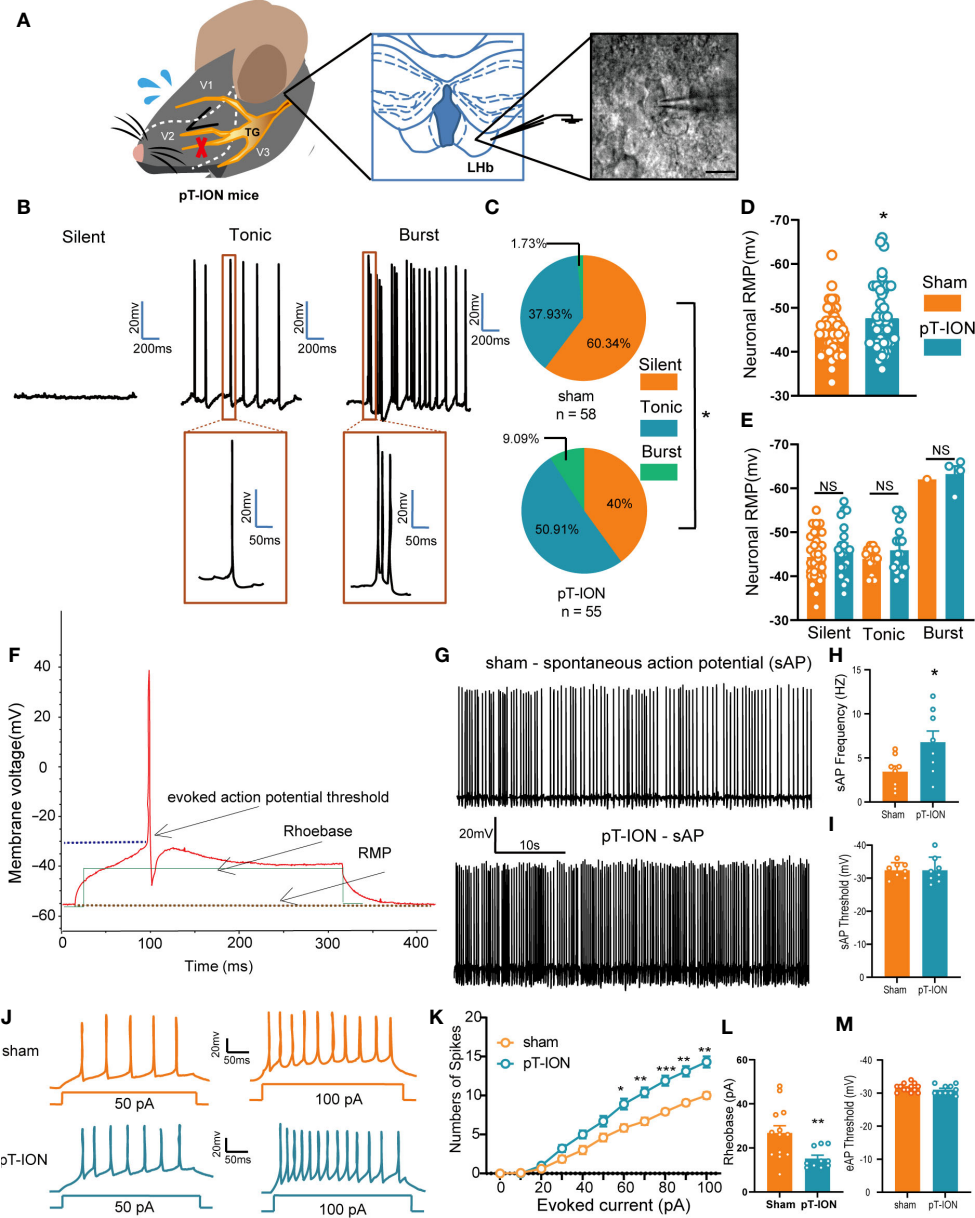
The LHb was activated in pT-ION mice. **(A)** Schematic of the trigeminal nerve anatomy and innervated region showing the infraorbital branch and the pT-ION ligation and transection point (left). Schematic of the whole-cell patch clamp recording in the LHb of pT-ION mice (middle). Sample image showing LHb neurons under clamp (right). Scale bar, 10 µm. **(B)** Representative traces of silent, tonic-firing, and burst-firing of LHb neurons. **(C)** Pie charts showing the percent abundance of the three types of LHb neurons in pT-ION and sham mice (*p < 0.05 by Chi squared test, n = 58 neurons from 11 mice in the sham group, n = 55 neurons from 12 mice in the pT-ION group). **(D)** The RMP recorded from LHb neurons of pT-ION and sham mice. **(E)** The RMP recorded from LHb neurons of pT-ION and sham mice and divided into three types: silent, tonic-firing, and burst-firing (n = 49 neurons from 10 mice in the sham group, n = 43 neurons from 10 mice in the pT-ION group). **(F)** Schematic of the action potential threshold, RMP, and rheobase (minimum current required to fire an action potential). **(G, H)** Typical traces **(G)** and data of spontaneous action potential firing spikes **(H)** recorded from LHb neurons of pT-ION and sham mice (*p < 0.05 vs. sham). **(I)** Data of spontaneous action potential thresholds recorded from LHb neurons of pT-ION and sham mice (n = 8 neurons from 8 mice). **(J)** Sample traces of evoked action potentials recorded from the LHb neurons of sham or pT-ION mice. **(K)** Firing spikes recorded from the LHb neurons of pT-ION and sham mice (*p < 0.05, **p < 0.01, ***p < 0.001 vs. control, n = 35 neurons from 8 mice in the control group, n = 28 neurons from 8 mice in the pT-ION group). **(L, M)** Data of rheobase **(L)** and evoked action potential threshold **(M)** recorded from LHb neurons of pT-ION and sham mice (**p < 0.01 vs. sham, n = 13 neurons from 8 mice in the sham group, n = 10 neurons from 8 mice in the pT-ION group).

In our previous work (19) we developed the orofacial neuropathic pain model in mice by pT-ION, and these mice exhibited allodynia in the V2 skin (dominated by the maxillary nerve of the trigeminal nerve) and V3 area (dominated by the mandibular nerve of the trigeminal nerve). In addition, pT-ION mice with trigeminal nerve injury exhibited anxiety-like behaviors in behavior tests. To test what happened in the LHb of pT-ION mice, we detected the neuronal activation-associated protein expression level using western blotting and analyzed the electrical activity of neurons using whole-cell patch clamp recording. The expression of phosphorylated Ca^2+^/calmodulin-dependent protein kinase II (p-CaMKII) and CaMKII was increased in pT-ION mice compared with the sham mice (**Figures S1A, B**) while the ratio of p-CaMKII/CaMKII remained unchanged (**Figure S1C**). pT-ION did not increase the expression of protein kinase A (PKA) protein but increased the expression of phosphorylated PKA (p-PKA) (**Figures S1D, E**). Also, the ratio of p-PKA/PKA showed an upward trend, but no significant difference was seen in the pT-ION group (**Figure S1F**). Electrophysiological recordings of isolated brain slices were used to record the action potential of the neurons of the LHb (**Figure 1A**). As shown previously (20), the firing patterns of LHb neurons are diverse and mainly include three types – silent, tonic-firing, and burst-firing (**Figure 1B**) – and the percentage of silent neurons in pT-ION mice was decreased while the percentage of tonic-firing and burst-firing neurons was increased (**Figure 1C**). When combining three types of neurons together, the resting membrane potentials of LHb neurons in pT-ION mice were on average lower than in sham mice (**Figure 1D**), indicating that LHb neurons in pT-ION mice were more hyperpolarized compared to sham mice. However, when separating the three types of neurons, the resting membrane potentials of silent neurons, tonic-firing neurons, and burst-firing neurons in pT-ION mice were unchanged compared with sham mice (**Figure 1E**), suggesting that the more hyperpolarized resting membrane potential (RMP) on average may be due to the increased proportion of burst-firing neurons after pT-ION. Compared with sham mice, the frequency of spontaneous action potential firing spikes of tonic-firing neurons in the LHb was increased (**Figures 1F–H**), while the threshold remained unchanged in pT-ION mice (**Figure 1I**). For silent neurons in the LHb, pT-ION increased the numbers of evoked action potential firing spikes (**Figures 1J, K**) and decreased the rheobase of silent neurons (**Figure 1L**) but did not change the threshold (**Figure 1M**). The above experimental results showed that LHb neurons were abnormally activated in pT-ION mice.

**Figure 2:**
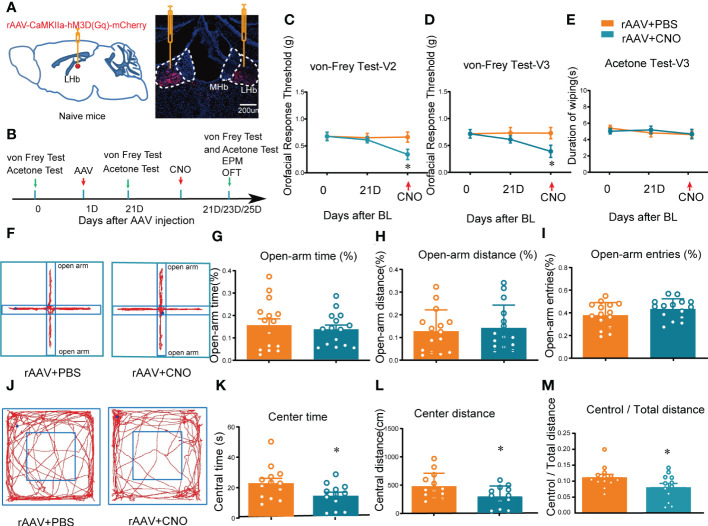
Mice showed orofacial allodynia and anxiety-like behaviors after bilateral activation of the LHb. **(A)** The injection sites of rAAV-CaMKIIa-hM3D(Gq)-mCherry-WPREs-pA virus (left) and typical immunofluorescence staining (right). Red, mCherry; Blue, DAPI. **(B)** The time schedule of behavioral testing, virus injection, and CNO injection. **(C, D)** Chemogenetic activation of bilateral LHb neurons induced allodynia in the orofacial V2 area **(C)** and the V3 skin **(D)** (*p < 0.05 vs. the WT+rAAV+PBS group). **(E)** Cold allodynia was unchanged in the V3 area as indicated by the lack of statistical differences in the duration of wiping in response to acetone stimulation. **(F)** Typical trajectory diagram of the EPM test. **(G-I)** The percentage of time **(G)**, distance **(H)**, and entries **(I)** in the open-arm of the EPM were unchanged by the activation of bilateral LHb neurons. **(J)** Typical trajectory diagram of the OFT. **(K-M)** The central time **(K)**, central distance **(L)**, and the central/total distance **(M)** in the OFT were reduced by the activation of bilateral LHb neurons by HM3D(Gq)-CNO (*p < 0.05 vs. the WT+rAAV+PBS group).

LHb neurons have been shown to be predominantly excitatory glutamatergic neurons, and accumulating evidence (21, 22), including our own results (19), suggests that the glutamatergic neurons in the LHb play key roles in orofacial and somatosensory conduction as well as anxiety-like behaviors in mice. However, whether the activation of LHb neurons in normal naïve mice has any effects on sensory conduction and anxiety has remained unknown. Here, we chemogenetically manipulated the LHb with hM3D(Gq)-CNO to mimic the activation of the bilateral LHb glutamatergic neurons. At 3 weeks after rAAV-CaMKIIa-hM3D(Gq)-mCherry injection into the LHb, CNO (2.5 mg/kg, i.p.) was administered (**Figures 2A, B**). The von Frey test and acetone test were used to evaluate mechanical allodynia and cold allodynia within the paw and orofacial area of the mice. Evaluation of mechanical allodynia considers that von Frey filament stimulation below the normal threshold can also cause abnormal responses, and cold allodynia was characterized by increased reactivity to cold stimuli applied by spraying acetone (23). The mice given CNO exhibited allodynia to mechanical stimuli in both the V2 and V3 skin areas (**Figures 2C, D**), while no difference was observed in the duration of wiping due to cold stimuli on the orofacial V3 area, compared to mice administered PBS (**Figure 2E**). The thresholds for mechanical stimulation and acetone application in the paw area were unchanged after CNO injection (**Figure S2**).

In addition, to determine whether mice with activation of LHb neurons exhibited anxiety-like behaviors, the mice were subjected to the EPM and OFT after CNO administration. These behavior tests showed that the percentage of open-arm distance, open-arm entries, and open-arm time in the EPM of mice with activation of bilateral LHb neurons remained unchanged compared to the control group (**Figures 2F–I**), indicating that chemogenetic activation of bilateral LHb glutamatergic neurons does not induce anxiety-like behaviors in the EPM. However, in the OFT the central time, central distance, and central/total distance (**Figures 2K–M**) were reduced by CNO administration, indicating that activation of bilateral LHb glutamatergic neurons in naive mice induced anxiety-like behaviors in the OFT. Together, these results suggested that orofacial allodynia but not paw allodynia and anxiety-like behaviors are associated with the abnormal excitation of LHb glutamatergic neurons.

### 3.2 Pharmacological activation of NK3R in the LHb attenuates orofacial allodynia and pain-related anxiety-like behaviors by suppressing the abnormal excitation of LHb neurons

**Figure 3:**
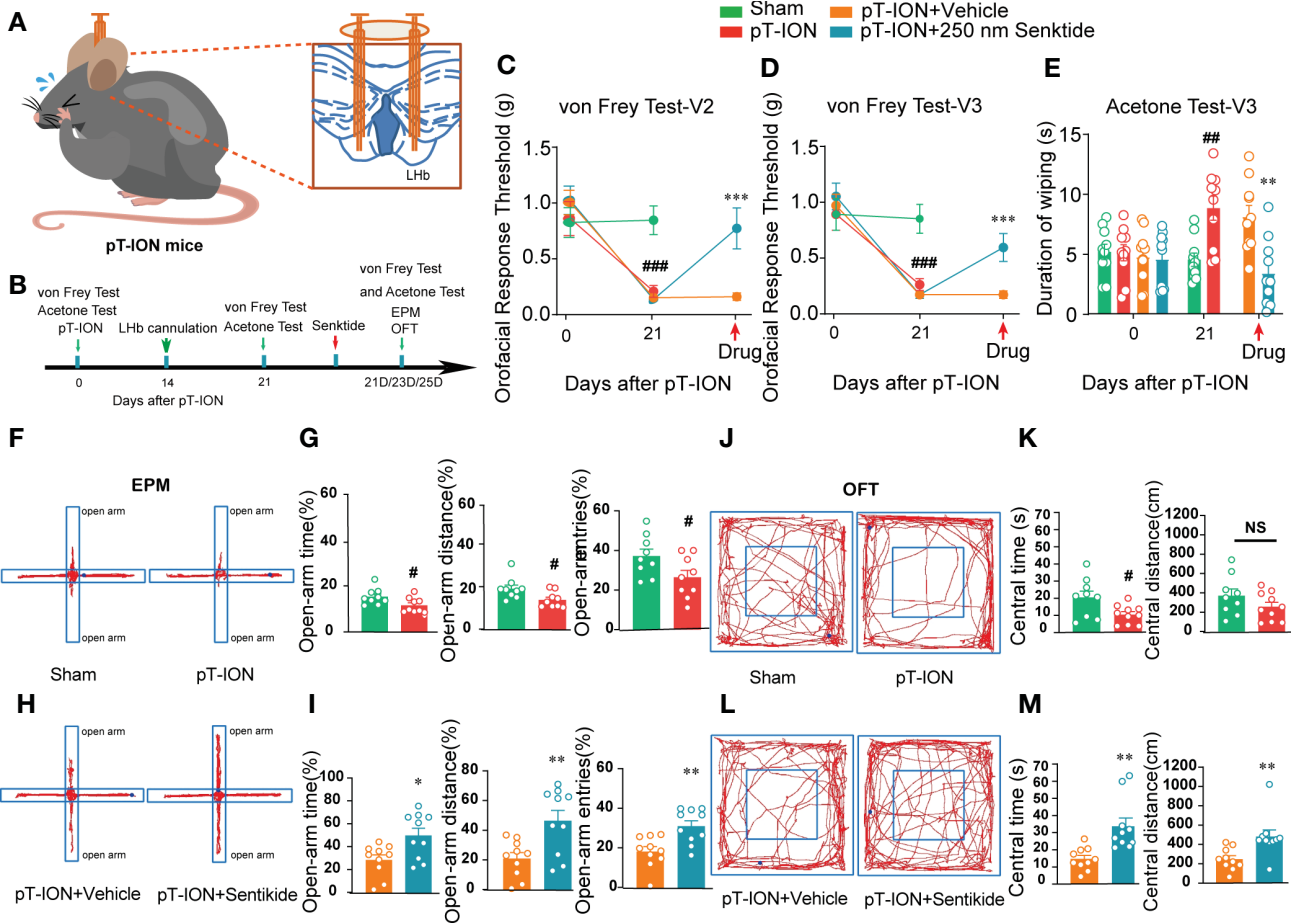
Bilateral injection of the NK3R agonist senktide into the LHb attenuated pT-ION-related allodynia and pain-associated anxiety-like behaviors. **(A)** Schematic of bilateral cannula implantation in the LHb of pT-ION mice. **(B)** The time schedule of the behavioral testing, pT-ION operation, cannulation, and drug administration. **(C–E)** pT-ION induced primary allodynia **(C)** and secondary allodynia **(D)**, as shown by reduced thresholds to mechanical stimuli, and secondary cold allodynia **(E)** was also induced in the V3 skin. The V2 and V3 mechanical allodynia and V3 cold allodynia were improved by bilateral senktide injection into the LHb (## p < 0.01, ###p < 0.001 vs. the sham group; **p < 0.01, *** p < 0.001 vs. the pT-ION+vehicle group; n = 10 mice per group). **(F, H)** Typical trajectories in the EPM. **(G)** The open-arm time, distance, and entries on day 21 after pT-ION were decreased (#p < 0.05, vs. the sham group, n = 10 mice per group). **(I)** The decreases in open-arm time, distance, and entries were completely reversed by bilateral senktide injection into the LHb (*p < 0.05, **p < 0.01 vs. the pT-ION+vehicle group, n = 10 mice per group). **(J, L)** Typical trajectories in the OFT. **(K)** The central times were decreased on day 21 after pT-ION while the central distance remained unchanged (#p < 0.05 vs. the sham group, n = 10 mice per group). **(M)** The central distance and central time were significantly increased by bilateral senktide injection into the LHb (**p < 0.01 vs. the pT-ION+vehicle group, n = 10 mice per group). NS, not significant.

NK3R has both analgesic and pain-inducing effects under different conditions (8), and it has been reported to have anxiolytic effects in rodents including mice and rats (14, 24). Nevertheless, whether and how NK3R activity in the LHb affects orofacial allodynia and pain-related anxiety-like behaviors is not clear. Here, we showed that compared with the control sham group, the expression of NK3R in the LHb was decreased after pT-ION (**Figure S3B**). To test whether deficiency of NK3R in the LHb was involved in orofacial allodynia and pain-related anxiety-like behaviors, we implanted a cannula for local senktide (a selective NK3R agonist) delivery to activate NK3R or for vehicle delivery as the control (**Figure S3A**, **Figure 3A**). Behavioral tests were performed on day 0 and day 21 after pT-ION or sham surgery (**Figure 3B**). Consistent with our previous results, the ipsilateral orofacial V2 area and V3 area showed decreased response thresholds after pT-ION on the 21st day after surgery (**Figures 3C, D**), and sham mice also showed cold allodynia over the same time (**Figure 3E**), indicating the occurrence of mechanical allodynia and cold allodynia after pT-ION. However, pharmacological activation of NK3R by delivery of senktide into the bilateral LHb reversed the decreased pain threshold induced by pT-ION compared with the vehicle delivery group (**Figures 3C, D**). Senktide delivery also attenuated the cold allodynia on the orofacial V3 area (**Figure 3E**), indicating that activation of NK3R reversed pT-ION-induced mechanical allodynia and secondary cold allodynia. Compared with the pT-ION+vehicle group, the pT-ION+senktide group showed increased exploration in the open-arm (**Figures 3H, I**). In addition, the central area exploration in the OFT was increased in the pT-ION+senktide group at 21 days (**Figures 3L, M**), while the total distance remained unchanged (**Figure S3C**). The performance of pT-ION+senktide mice in both the EPM and OFT suggested that activating NK3R in the LHb alleviated the orofacial allodynia-induced anxiety-like behaviors.

**Figure 4:**
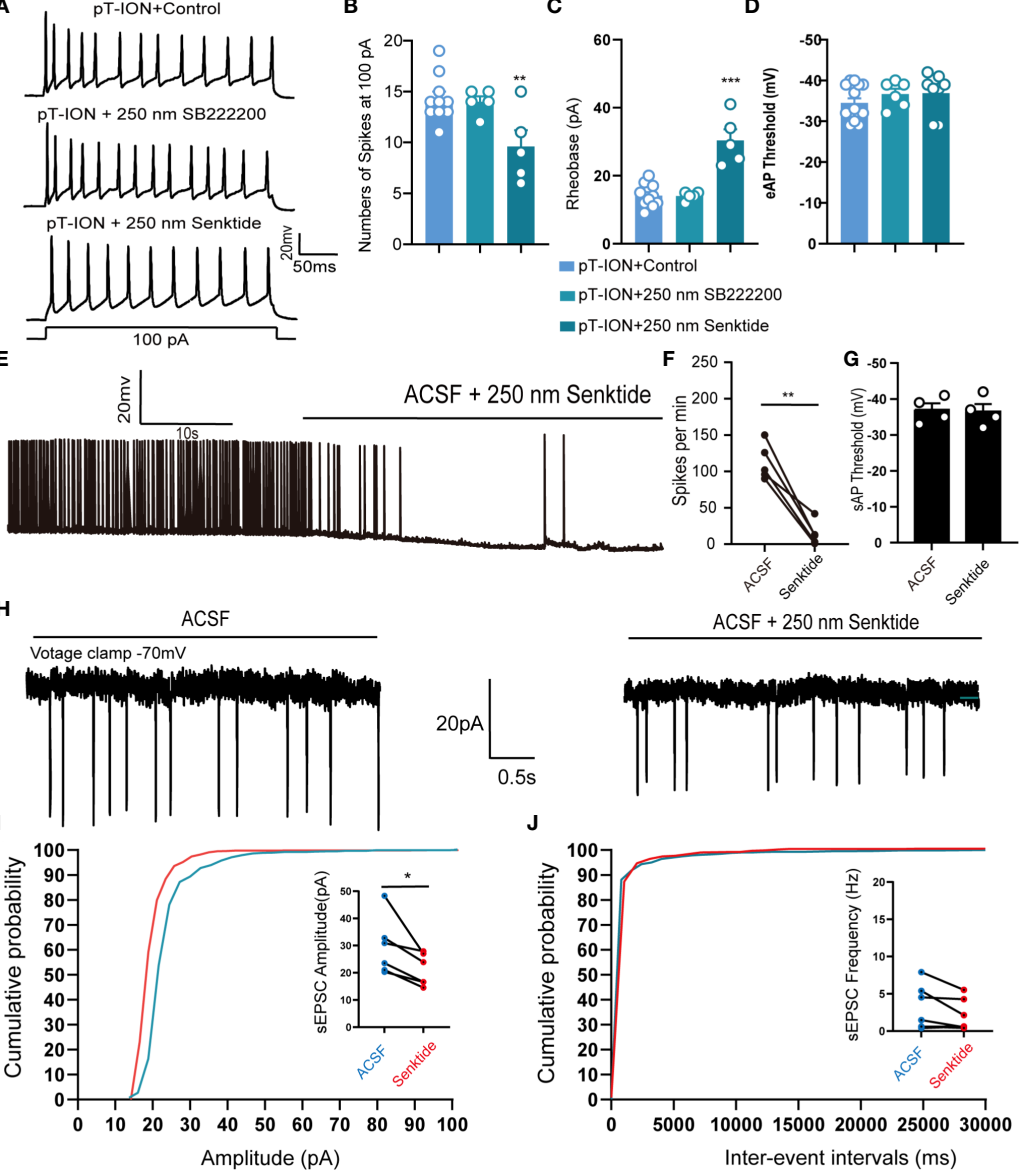
Pharmacological activation of NK3R in the LHb suppressed the pT-ION-induced abnormal excitation of neurons. **(A)** Patch-clamp recording showing the typical traces recorded from LHb neurons with SB222200 and senktide perfusion in pT-ION mice. **(B–D)** Data of evoked action potential firing spikes at 100 pA current stimulation **(B)**, rheobase **(C)**, and evoked action potential threshold **(D)** recorded from LHb neurons with SB222200 and senktide perfusion in pT-ION mice (**p < 0.01 vs. pT-ION+control, ***p < 0.001 vs. pT-ION+control, n = 10 neurons from 5 mice in the control group, n = 5 neurons from 5 mice in the SB222200 group, n = 5 neurons from 5 mice in the senktide group). **(E)** Typical traces of spontaneous action potential firing spikes recorded from LHb neurons with senktide perfusion in pT-ION mice. **(F, G)** Data of spontaneous action potential firing spikes **(F)** and spontaneous action potential thresholds **(G)** recorded from LHb neurons with senktide perfusion in pT-ION mice (**p < 0.01 vs. artificial cerebrospinal fluid, n = 5 neurons from 5 mice). **(H)** Typical frequency and amplitude of sEPSCs of LHb neurons with senktide perfusion in pT-ION mice. **(I, J)** Corresponding cumulative distributions and quantification of sEPSC amplitudes **(I)** and frequency **(J)** of LHb neurons with senktide perfusion in pT-ION mice (*p < 0.05 vs. artificial cerebrospinal fluid, n = 6 neurons from 4 mice).

We thus were curious about the mechanism through which activation of NK3R might affect pT-ION-induced excitation of neurons in the LHb. Ex vivo electrophysiology was used to record the spontaneous action potential firing of tonic firing neurons and evoked action potential firing of silent neurons in the LHb. Pharmacological activation of NK3R by senktide decreased the numbers of evoked action potential firing spikes (**Figures 4A, B**) and increased the rheobase (**Figure 4C**) compared with artificial cerebrospinal fluid perfusion in mouse brain slices after pT-ION, while inhibition of NK3R by the NK3R antagonist SB-222200 had no effect on the number of evoked action potential firing spikes. Neither senktide nor SB-222200 changed the evoked action potential threshold (**Figure 4D**). We then recorded the spontaneous action potential firing to verify the role of the NK3R agonist senktide on LHb neurons, and we found that activation of NK3R decreased the frequency of spontaneous action potential firing spikes (**Figures 4E, F**) but did not change the action potential threshold (**Figure 4G**). To explore if synaptic transmission participates in the effect of senktide on LHb neurons, we recorded spontaneous excitatory postsynaptic currents (sEPSCs) in the LHb. The cumulative probability curve of the amplitude of sEPSCs was left-shifted by senktide, indicating the inhibition of the amplitude of sEPSCs by the activation of NK3R (**Figure 4I**). The cumulative probability curve of the inter-event intervals of sEPSC was not left- or right-shifted, indicating no effects of senktide on the frequency of sEPSCs (**Figure 4J**). Thus, the whole-cell patch clamp recording data suggest that LHb neurons were activated after pT-ION and that pharmacological activation of NK3R in the LHb neurons could suppress the abnormal excitation, thus indicating that pharmacological activation of NK3R in the LHb neurons reversed pT-ION-induced orofacial allodynia and pain associated anxiety-like behaviors by suppressing the abnormal excitation of LHb neurons.

### 3.3 Pharmacological inhibition of NK3R in the LHb caused orofacial allodynia and anxiety-like behaviors in naive mice by increasing neuronal excitability

**Figure 5:**
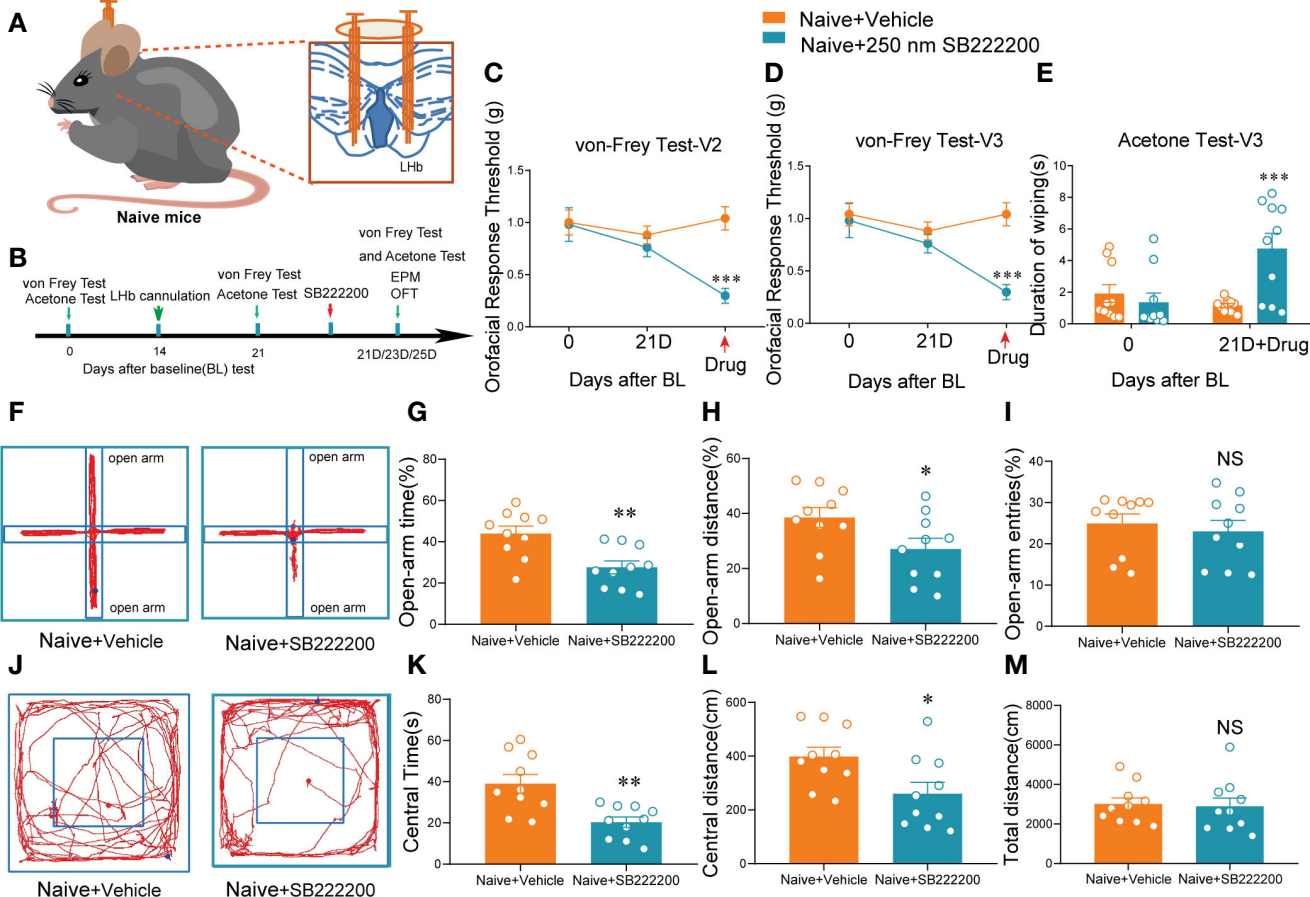
Bilateral NK3R antagonist SB222200 injection into the LHb induced orofacial allodynia and anxiety-like behaviors in naive mice. **(A)** Schematic of bilateral cannula implantation in the LHb of naive mice. **(B)** The time schedule of behavioral testing, cannulation, and drug administration. **(C–E)** Bilateral SB222200 injection into the LHb induced allodynia in the V2 skin **(C)** and V3 skin **(D)** and secondary cold allodynia in the V3 skin **(E)** (***p < 0.001 vs. the Naive+vehicle group, n = 10 mice per group). **(F)** Typical trajectories in the EPM. **(G–I)** Bilateral SB222200 injection into the LHb decreased the percent of open-arm time **(G)**, distance **(H)**, and entries **(I)** (*p < 0.05, **p < 0.01, vs. the Naive+vehicle group, n = 10 mice per group). **(J)** Typical trajectories in the OFT. **(K–M)** Bilateral SB222200 injection into the LHb decreased the central time **(K)** and central distance **(L)** while the total distance **(M)** remained the same (*p < 0.05, **p < 0.01, vs. the Naive+vehicle group, n = 10 mice per group).

Given that the expression of NK3R was decreased after pT-ION vs. the control group (**Figure S3B**), we hypothesized that pharmacological inhibition of NK3R in the LHb would affect orofacial sensation in naive mice. To test this hypothesis, we implanted a cannula for local SB-222200 or vehicle delivery into the LHb (**Figure 5A**). The same as before, the mice were given 1 week to recover after transplantation before performing the behavioral tests (**Figure 5B**). The orofacial V2 area response thresholds of the mice with SB-222200 delivery were significantly decreased (**Figure 5C**), while the V3 nociceptive response was also decreased (**Figure 5D**). The V3 area also showed cold allodynia (**Figure 5E**). Of note, behavior tests showed that after delivery of SB-222200 to the bilateral LHb the mice had a lower percentage of open-arm time and distance in the EPM, but not a reduction in open-arm entries (**Figures 5F–I**). In addition, after delivery of SB-222200 to the bilateral LHb, mice explored less in the central area of the OFT, while the total distance remained unchanged compared with vehicle delivery (**Figures 5J-M**). Together, these results suggest that pharmacological inhibition of NK3R by SB-222200 in the LHb induced orofacial allodynia and anxiety-like behaviors in naive mice.

**Figure 6:**
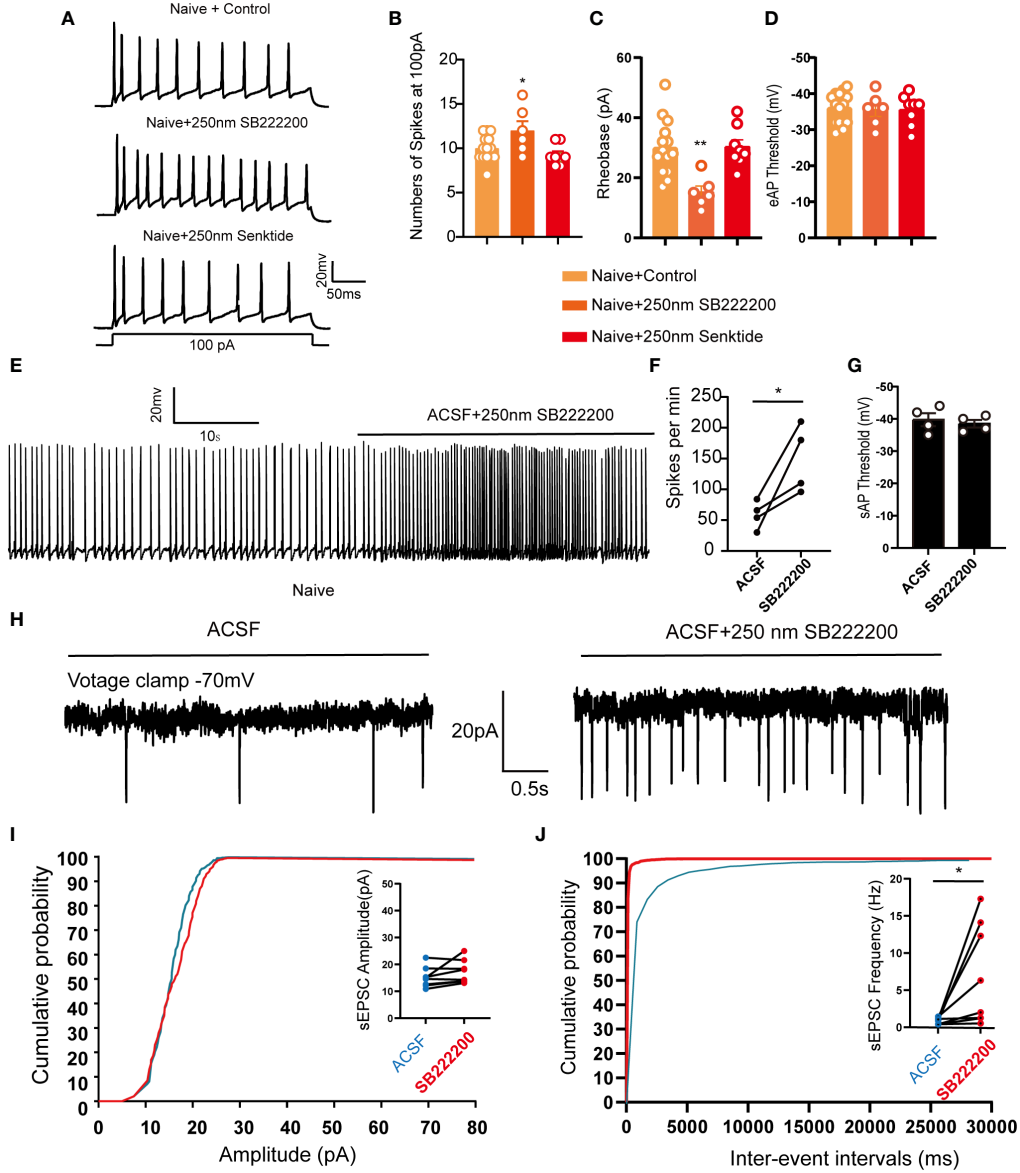
LHb neurons were activated after pharmacological inhibition of NK3R in naive mice. **(A)** Patch-clamp recording showing the typical traces recorded from LHb neurons with SB222200 and senktide perfusion in naive mice. **(B)** Data of evoked action potential firing spikes at 100 pA current stimulation recorded from LHb neurons with SB222200 and senktide perfusion in naive mice (*p < 0.05 vs. the Naive+control group, n = 12 neurons in the control group from 6 mice, n = 6 neurons from 6 mice in the SB222200 group, n = 7 neurons from 7 mice in the senktide group). **(C, D)** Data of rheobase **(C)** and evoked action potential thresholds **(D)** recorded from LHb neurons with SB222200 and senktide perfusion in naive mice (**p < 0.01 vs. the Naive+control group, n = 14 neurons in the control group from 6 mice, n = 6 neurons from 6 mice in the SB222200 group, n = 8 neurons from 7 mice in the senktide group). **(E)** Typical traces of spontaneous action potential firing spikes recorded from LHb neurons with SB222200 perfusion in naive mice. **(F, G)** Data of spontaneous action potential firing spikes **(F)** and spontaneous action potential thresholds **(G)** recorded from LHb neurons with SB222200 perfusion in naive mice (*p < 0.05 vs. artificial cerebrospinal fluid, n = 4 neurons from 4 mice). **(H)** Typical frequency and amplitude of sEPSCs of LHb neurons with SB222200 perfusion in naive mice. **(I, J)** Corresponding cumulative distributions and quantification of sEPSC amplitudes **(I)** and frequency **(J)** of LHb neurons with SB222200 perfusion in naive mice (*p < 0.05 vs. artificial cerebrospinal fluid, n = 8 neurons from 4 mice).

We previously showed that activation of bilateral LHb glutaminergic neurons in naive mice could induce orofacial allodynia (**Figure 2**), and we hypothesized that inhibition of NK3R in the LHb also affects the activity of neurons. Whole-cell patch clamp showed that compared with artificial cerebrospinal fluid perfusion in mouse brain slices, pharmacological activation of NK3R by senktide had no effect on the number of evoked action potential firing spikes or the rheobase, while inhibition of NK3R by SB-222200 increased the numbers of evoked action potential firing spikes and decreased the rheobase (**Figures 6A–C**). However, senktide perfusion or SB-222200 perfusion did not change the evoked threshold of silent neurons (**Figure 6D**). We further verified the role of the NK3R antagonist SB-222200 on LHb neurons by recording their spontaneous action potential firing, and not surprisingly inhibition of NK3R could increase the frequency of spontaneous action potential firing spikes (**Figures 6E, F**) while it did not change the spontaneous action potential threshold (**Figure 6G**). The cumulative probability curve of the inter-event intervals of sEPSC was left-shifted with no changes in the cumulative probability curve of the amplitude, indicating the increase in the frequency but not the amplitude of sEPSCs by the blockade of NK3R (**Figures 6I, J**). The above experimental results showed that LHb neurons were activated after pharmacological inhibition of NK3R.

Activation of neurons can also be reflected by neuronal calcium influx, so we cultured primary hippocampal neurons to verify that NK3R is involved in calcium influx in neurons. Consistent with the electrophysiological results, compared with controls pharmacological activation of NK3R by senktide had no effect on calcium influx, while inhibition of NK3R by SB-222200 could induce calcium influx in neurons (**Figures S4A–C**). Together, these results indicated that pharmacological inhibition of NK3R could activate neurons.

### 3.4 The fPAG projects NKB-positive nerve fibers to the LHb

**Figure 7:**
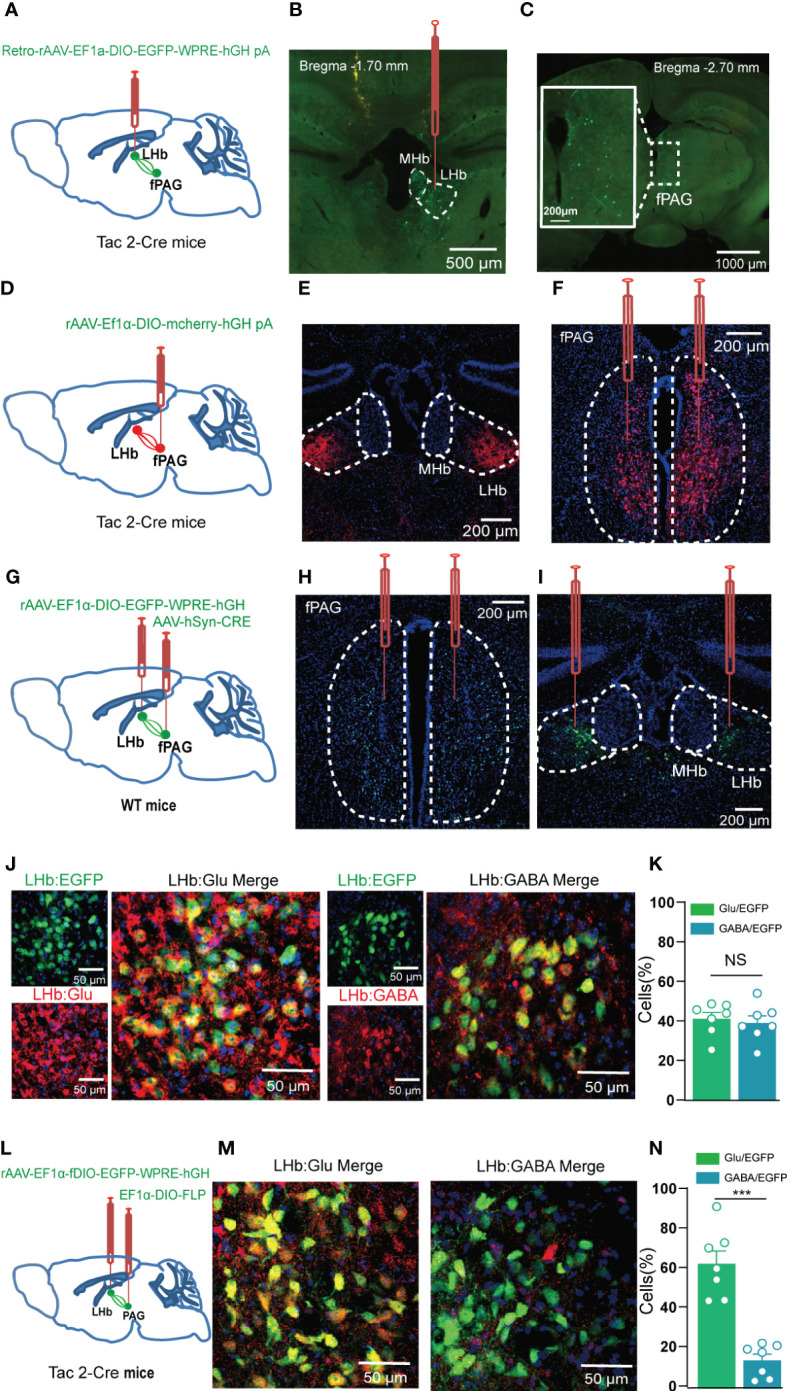
The fPAG projects NKB-positive nerve fibers to the LHb. **(A, B)** Schematic **(A)** and representative image **(B)** of injection of Retro-rAAV-EF1a-DIO–EGFP into the LHb of Tac2-Cre mice. **(C)** Representative image of fPAG EGFP^+^ neurons. **(D)** Schematic of injection of anterograde rAAV-EF1a-DIO–mCherry-hGH into the fPAG of Tac2-Cre mice. **(E)** Representative image of an mCherry+ terminals in the LHb. **(F)** Typical image of fPAG neurons. **(G)** Schematic of the Cre-dependent anterograde trans-monosynaptic tracing strategy in WT mice. **(H, I)** Typical images showing the neurons in the fPAG **(H)** and the LHb **(I)**. **(J)** Images showing that GFP-labeled neurons within the LHb co-localized with glutamate and GABA immunofluorescence. **(K)** Data showing that GFP-labeled neurons within the LHb co-localized with glutamate and GABA immunofluorescence. **(L)** Schematic of the Cre-dependent anterograde trans-monosynaptic tracing strategy in Tac2-Cre mice. **(M)** Images showing that GFP-labeled neurons within the LHb of Tac2-Cre mice co-localized with glutamate and GABA immunofluorescence. **(N)** Data showing that GFP-labeled neurons within the LHb mainly co-localized with glutamate immunofluorescence in Tac2-Cre mice. ***p<0.001.

As a typical G-protein–coupled receptor, NK3R functions by binding to NKB, which is one of the members of the tachykinin family and is encoded by the *Tac2* gene (8). To look for upstream sources of NKB release into the LHb, retrograde Cre-dependent viral labeling (Retro-rAAV-EF1a-DIO–EGFP) was used in the Tac2-Cre mouse line in which Cre is expressed under control of the *Tac2* gene in NKB^+^ neurons (**Figure 7A**). Whole-brain images were taken to track the regions that had been labeled by the LHb-injected retrovirus, and multiple regions were found to have NKB^+^ projections into the LHb, including the lateral hypothalamic area, the medial amygdaloid nucleus, and the fPAG (**Figures S5A–C**). We focused on the fPAG at bregma –2.70 mm, which was identified as one of the regions most enriched in NKB^+^ projections to the LHb (**Figures 7B, C**). In order to verify that the fPAG does indeed project NKB^+^ neurons to the LHb, anterograde Cre-dependent virus tracing (rAAV-EF1a-DIO–mCherry-hGH) was used in the LHb of Tac2-Cre mice (**Figure 7D**). Distinct mCherry-labeled nerve fibers were observed in the LHb 3 weeks after rAAV-EF1a-DIO–mCherry was injected into the bilateral fPAG (**Figures 7E, F**). Together, these results indicate that NKB^+^ neurons project from the fPAG to the LHb.

**Figure 8:**
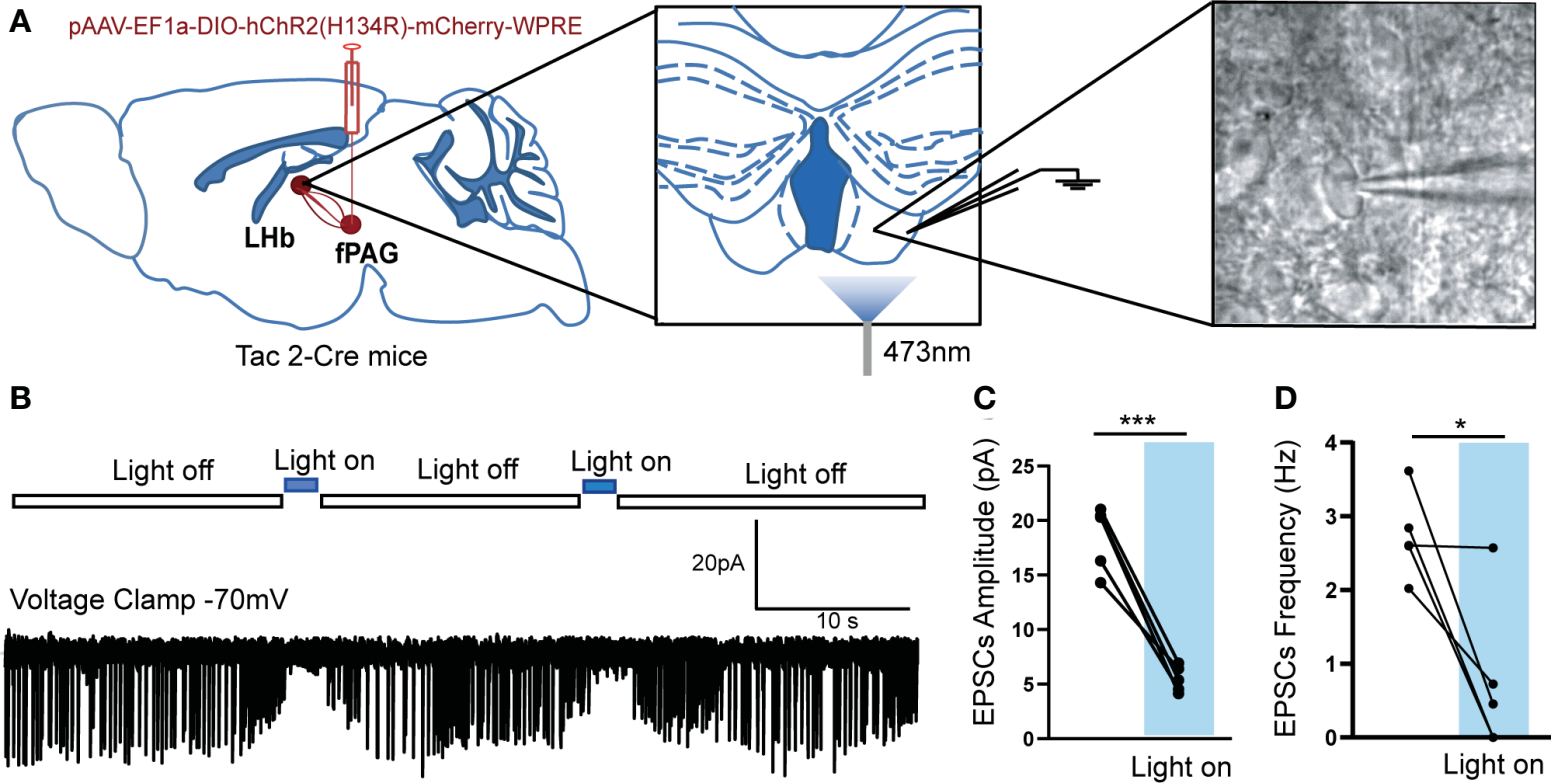
Optogenetically evoked EPSCs in LHb neurons projected from fPAG NKB-positive neurons. **(A)** Schematic of pAAV-EF1a-DIO-hChR2(H134R)-mCherry-WPRE injection into fPAG, a 470-nm laser application, and the recording configuration in the LHb. **(B)** Representative traces of light-evoked EPSCs recorded in LHb neurons in Tac2-cre mice. **(C, D)** Quantification of EPSC amplitudes **(C)** and frequencies **(D)** of LHb neurons with laser application in Tac2-cre mice (*p< 0.05, ***p < 0.001 vs. light off, n = 5 neurons from 3 mice).

To further confirm the fPAG–LHb projection, we injected anterograde trans-monosynaptic rAAV-hSyn-CRE-EGFP virus into the fPAG and rAAV-EF1α-DIO-EGFP virus into the LHb of wildtype C57 mice (**Figures 7G–I**). Three weeks later, neurons positive for green fluorescent protein (GFP^+^) were observed in the LHb (**Figure 7I**), and these were co-localized with both a glutamate-specific antibody (~40%) and a GABA-specific antibody (~40%; **Figures 7J, K**). Of note, the results of the Cre-dependent anterograde trans-monosynaptic tracing strategy in WT mice only indicated that nonspecific projected neurons from the fPAG to the LHb had synaptic connections with both glutamatergic and GABAergic neurons in the LHb. To determine what kinds of neurons the NKB^+^ projections from the fPAG to the LHb have synaptic connections with, we injected anterograde trans-monosynaptic rAAV- EF1α-DIO-FLP virus into the fPAG and rAAV-EF1α-fDIO-EGFP virus into the LHb of Tac2-Cre mice (**Figure 7L**). Three weeks later, GFP^+^ neurons in the LHb were co-localized with both a glutamate-specific antibody (~60%) and a GABA-specific antibody (~10%; **Figures 7M, N**), suggesting that fPAG^NKB^ → LHb involves synaptic connections mostly with glutamatergic neurons and not with GABAergic neurons. To further characterize the functional connections in this fPAG^NKB^ → LHb pathway, we used optogenetics combined with electrophysiology recordings (**Figure 8A**). The 473 nm light stimulation of ChR2-containing NKB-positive fPAG terminals in the LHb decreased the amplitude and frequency of EPSCs (**Figures 8B-D**). Taken together, these data suggest that NKB-positive neurons in the fPAG send functional projections to the LHb.

### 3.5 fPAG^NKB^ → LHb regulated orofacial allodynia and anxiety-like behaviors in pT-ION mice

**Figure 9:**
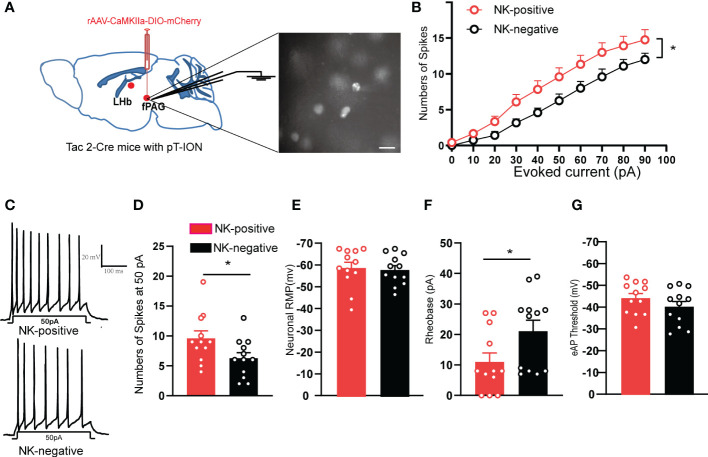
The fPAG NKB-positive neurons were active in pT-ION mice. **(A)** Schematic of rAAV-CaMKIIa-DIO-mCherry injection and the recording configuration in the fPAG (left). Sample image showing NKB-positive neurons labelled with mCherry through virus injection into the fPAG of Tac2-Cre mice (right). Scale bar, 10 µm. **(B)** Firing spikes recorded from the fPAG neurons of NKB-positive and NKB-negative neurons of pT-ION Tac2-cre mice. **(C)** Sample traces of evoked action potentials at 50 pA current stimulation recorded from NKB-positive and NKB-negative neurons in the fPAG. **(D–G)** Data of evoked action potential firing spikes at 50 pA current stimulation **(D)**, RMP **(E)**, rheobase **(F)**, and evoked action potential threshold **(G)** recorded from NKB-positive and NKB-negative neurons in the fPAG (*p < 0.05 vs. NKB-negative neurons, n = 12 neurons in NKB-positive and n = 12 neurons in NKB- negative from 5 mice).

To test the intrinsic neuronal properties of glutamatergic NKB-positive neurons that project from the fPAG to the LHb, the evoked action potential of glutamatergic NKB-positive neurons and NKB-negative neurons in the fPAG after pT-ION were recorded (**Figure 9A**). Compared with NKB-negative neurons, NKB-positive neurons had greater numbers of evoked action potential spikes (**Figures 9B-D**) and lower rheobase (**Figure 9F**), while the resting membrane potential (**Figure 9E**) and threshold remained unchanged (**Figure 9G**). These data suggested the glutamatergic NKB-positive neurons were more active than NKB-negative neurons in the fPAG after pT-ION.

**Figure 10:**
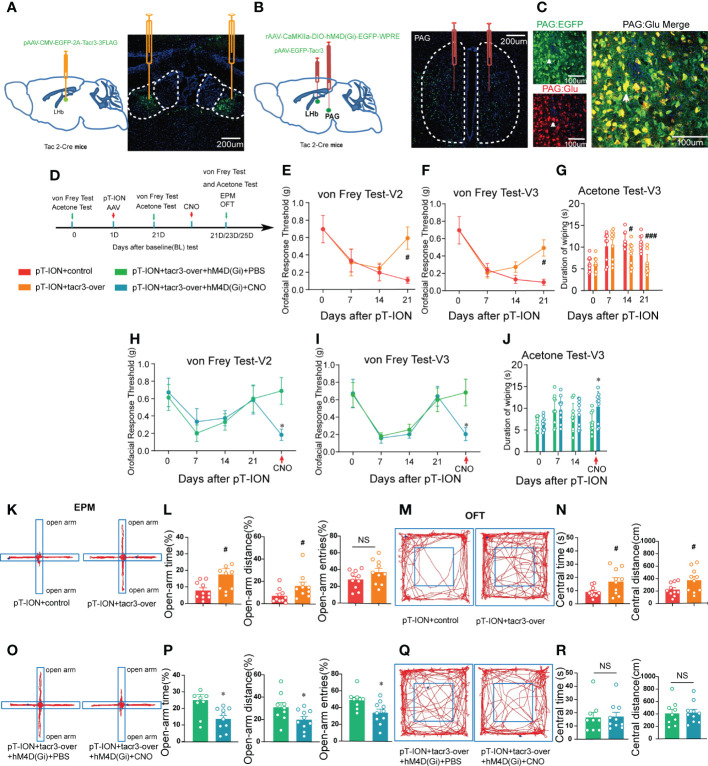
Inhibition of the release of NKB in the fPAG reversed the alleviating effect of *Tacr3* overexpression in the LHb on pT-ION-induced orofacial allodynia and pain-associated anxiety-like behaviors. **(A)** Schematic (left) and representative image (right) of the injection of pAAV-CMV-EGFP-2A-Tacr3-3FLAG into the LHb of Tac2-Cre mice. **(B)** Schematic of the injection of pAAV-CMV-EGFP-2A-Tacr3-3FLAG into the LHb and injection of rAAV-CaMKIIa-DIO-hM4D(Gi)-EGFP-WPRE-hGH into the fPAG of Tac2-Cre mice (left). Typical image of rAAV-CaMKIIa-DIO-hM4D(Gi)-EGFP-WPRE-hGH injection into the fPAG of Tac2-Cre mice (right). Green, EGFP; Blue, DAPI. **(C)** Images showing that GFP-labeled neurons within the fPAG co-localized with glutamate. **(D)** The time schedule of behavioral testing, pT-ION operation, virus injection, and CNO administration. **(E-G)** pT-ION–induced V2 allodynia **(E)**, V3 allodynia **(F)**, and V3 cold allodynia **(G)** were improved by bilateral pAAV-EGFP-Tacr3 injection into the LHb (#p < 0.05, ###p < 0.001 vs. the pT-ION+vehicle group, n = 10 mice per group). However, the improvements in primary mechanical allodynia **(H)**, secondary mechanical allodynia **(I)**, and secondary cold allodynia **(J)** were reversed by bilateral rAAV-CaMKIIa-DIO-hM4D(Gi)-EGFP injection into the fPAG after CNO administration. (*p < 0.05 vs. the pT-ION+tacr3-over+hM4D(Gi)+PBS group, n = 10 mice per group). **(K, O)** Typical trajectories in the EPM. **(L)** The percentages of open-arm time and distance after pT-ION were increased by bilateral pAAV-EGFP-Tacr3 injection into the LHb, while the percentage of open-arm entries remained unchanged (#p < 0.05 vs. the pT-ION+vehicle group, n = 10 mice per group). **(P)** The increased percentages of open-arm time, distance, and entries was reversed by bilateral rAAV-CaMKIIa-DIO-hM4D(Gi)-EGFP injection into the fPAG after CNO administration (*p < 0.05 vs. the pT-ION+tacr3-over+hM4D(Gi)+PBS group, n = 10 mice per group). **(M, Q)** Typical trajectories in the OFT. **(N)** The central distance and central time were increased by bilateral pAAV-EGFP-Tacr3 injection into the LHb (#p < 0.05 vs. the pT-ION+vehicle group, n = 10 mice per group). **(R)** The central distance and central time remained unchanged after bilateral rAAV-CaMKIIa-DIO-hM4D(Gi)-EGFP injection into the fPAG after CNO administration (n = 10 mice per group).

NK3R is encoded by the *Tacr3* gene (25). In our previous work, microarray analysis showed that the expression of *Tacr3* was downregulated in the LHb of pT-ION mice (19), and we confirmed here that reversing the downregulation of *Tacr3* in the bilateral LHb alleviated pT-ION-induced orofacial allodynia (**Figures 10E–G**) and pain-related anxiety-like behaviors (**Figures 10K–N**; **Figure S6A**). Here, we aimed to inhibit NKB^+^ neurons from releasing NKB from the fPAG to the LHb in order to test whether NKB release from the fPAG would affect the alleviating effect of *Tacr3* overexpression in the LHb on pT-ION-induced allodynia and anxiety-like behavior. The long-range projecting neurons from the PAG are predominantly excitatory pyramidal neurons and are glutamatergic, and these also play important roles in the neural circuits mediating anxiety-like responses to threats (26, 27). Therefore, we used chemogenetic inhibition of NKB^+^ glutamatergic neurons to inhibit the release of NKB from the fPAG to the LHb. On the day of the pT-ION model surgery, we injected pAAV-CMV-EGFP-2A-*Tacr3* into the bilateral LHb and the Cre-dependent chemogenetic inhibitory virus rAAV-CaMKIIa-DIO-hM4D(Gi)-EGFP into the bilateral fPAG of Tac2-Cre mice (**Figures 10A, B**), and behavior tests were performed 3 weeks later (**Figure 10D**). As shown in immunofluorescence images, there was significant colocalization of GFP^+^ neurons and glutamate-specific antibodies in the fPAG (**Figure 10C**). Compared with the pT-ION+*tacr3*-over+hM4D(Gi)+PBS group, pT-ION-induced V2 and V3 mechanical allodynia and V3 cold allodynia reappeared after CNO administration (**Figures 10H–J**). In addition, although the central time and central distance in the OFT remained unchanged by CNO administration in the pT-ION+*tacr3*-over+hM4D(Gi)+CNO group (**Figures 10Q, R**; **Figure S6B**), the open-arm time, open-arm distance, and open-arm entries in the EPM were decreased (**Figures 10O, P**), indicating that pT-ION-induced anxiety-like behaviors reappeared after the release of NKB in the fPAG was inhibited. Furthermore, there was no significant difference between male and female Tac2-cre mice in either painful or anxiety-like behaviors (**Figures S7** and **S8**). These experiments confirmed that chemogenetic inhibition of NKB release from the fPAG reversed the analgesic and anxiolytic effects of *Tacr3* overexpression in pT-ION mice.

## 4 Discussion

Trigeminal neuralgia is often chronic and is one of the most severe forms of chronic pain (28), and it is associated with a high risk of developing mental disorders (29). There are currently no effective treatments because the underlying mechanism of such pain and subsequent anxiety are not fully understood. In this study, we confirm the hypothesis that NK3R in the LHb mediates orofacial allodynia and anxiety-like behaviors by interfering with the excitability of neurons. The major findings of this study include the following. (1) Orofacial allodynia and pain-associated anxiety-like behavior were associated with abnormal excitation of LHb neurons. (2) Pharmacological activation of NK3R in the LHb neurons suppressed the abnormal excitation of LHb neurons in pT-ION mice and reversed orofacial allodynia and pain-related anxiety. (3) Pharmacological inhibition of NK3R activated LHb neurons and induced orofacial allodynia and anxiety-like behaviors in naive mice. (4) fPAG^NKB^→LHb regulated orofacial allodynia and pain-induced anxious behavior.

The LHb mediates negative emotions and is most widely studied in cases of depression mainly due to its abnormal excitation. Indeed, anxiety and depression are often concomitant (30), and trigeminal neuralgia mice show both anxiety and depression after model establishment (19, 31). The LHb is the only brain region that is observed to be abnormally hyperactive in depressed animals (32). Here, we showed that LHb neurons were also abnormally activated in pT-ION mice and that these mice had performance anxiety. Increasing β-CaMKII in the LHb has been shown to strongly enhance the synaptic efficacy and spike output of LHb neurons and to produce profound depressive symptoms, including anhedonia and behavioral despair in WT mice and rats (33). Our results showed that the RMPs of LHb neurons in pT-ION mice were on average lower than in sham mice. A previous study indicated that burst-firing neurons had more hyperpolarized RMPs than silent or tonic-firing neurons in the LHb (20). Our data showed that the increased RMP in the pT-ION group was not due to RMP changes in a specific cell types but to change in the percentage of different cell types. In the current study, we observed that activation of bilateral LHb neurons induced anxiety-like behaviors in naive mice. In contrast, pharmacological inhibition of NK3R in the LHb induced anxiety-like behaviors in naive mice by activating neurons in the LHb, which indicated that enhanced LHb activity could lead to depressive-like or anxiety-like effects in healthy naive rodents.

Evidence suggests that the LHb has a potential role in the processing of pain and analgesic signals in animals, and the blood flow to the habenula is significantly increased in rat models of chronic pain (34). In addition, a clinical MRI study of healthy volunteers showed that noxious thermal stimulation significantly enhanced habenula activity (35) and nociceptive stimulation increased the expression of c-fos protein in the habenula (36). Furthermore, LHb neurons showed excitatory respond to peripheral noxious stimuli (37). Based on the above evidence, we hypothesized that activation of the LHb neurons induces allodynia. Consistent with this hypothesis, our results showed that chemogenetic activation of bilateral glutamatergic LHb neurons induced allodynia. However, activation of the LHb neurons only induced orofacial allodynia and not paw allodynia. The LHb accepts both ascending pain-transmitting fiber projections from the trigeminal nucleus and spinal dorsal horn (38). The LHb has been reported to receive direct afferent inputs originating from lamina I of the dorsal horn, suggesting that painful signals can directly transmit to the LHb (39). Electrophysiological recordings have confirmed that LHb neurons are activated following noxious stimuli (40). Meanwhile, the LHb projects to the PAG and DRN (41, 42), which innervate dorsal horn neurons directly or indirectly *via* a descending inhibitory pathway and produce analgesia. Therefore, we did not find the decrease in the paw withdrawal threshold after chemogenetic activation of LHb neurons to be due to the activation of descending inhibition. Meanwhile, there is a difference in the neural pathway and circuitry for the processing of facial versus bodily pain. Fan Wang’s group revealed a direct monosynaptic connection between cranial sensory neurons and nociceptive neurons in the lateral parabrachial nucleus, which gives explanations for why the craniofacial pain is more severe than body pain (43). Therefore, the orofacial pain may be less affected by the descending inhibition that is activated upon chemogenetic activation of the LHb, and thus we observed significant facial allodynia.

Our results not only showed that chemogenetic activation of the LHb glutamatergic neurons could induce orofacial allodynia, but also demonstrated that activation of the LHb neurons by pharmacological inhibition of NK3R induced orofacial allodynia. In the classification of causes of pain, allodynia caused by intervention in the LHb belongs to central pain syndrome. Central pain syndrome mainly involves damage to the central nervous system (including the brain) (44). The phenotype of pain caused by central pain syndrome varies greatly in different individuals, mainly due to different etiologies (45). Central pain syndrome is characterized by a mixture of pain sensations, including constant burning sensations and mechanical allodynia. Also, the pain increases with temperature changes, most often cold temperatures (46, 47). Here, our results showed that pharmacological inhibition of NK3R in the LHb in mice resulted in both mechanical allodynia and cold allodynia, which is consistent with the general phenomenon of central pain syndrome (48). However, mice with chemogenetic activation of the LHb neurons exhibited mechanical allodynia but not cold allodynia. The mechanism of cold allodynia in central pain syndrome is complex and poorly studied. However, previous studies reported cold allodynia in patients with central pain syndrome, and it has been proposed that these patients have disruption of a thermosensory area in the insular cortex or activation of the anterior cingulate cortex (49, 50). The reason that cold allodynia disappears with chemogenetic activation of the LHb neurons might also be a potential effect of the chemogenetic method, and this should be studied further.

The EPM and OFT are routinely used to study anxiety-related behavior in mice by exploiting the natural aversion of rodents to exposed fields (51). In the EPM, rodents perceived as anxious are more likely to remain in the darkened, enclosed regions as opposed to exploring the brighter, open, and elevated regions of the apparatus (52). In the OFT, rodents tend to avoid the central unprotected area and to concentrate their movements near the walls, and the time exploring the central region is used as an anxiety-related index (53). Because anxiety-related emotionality is multidimensional (54), each test produces its own anxiety-related axis and measures different aspects of anxiety. It has been shown that emotionality-related behaviors from the OFT and EPM are loaded on distinct factors, thus reflecting different behavioral dimensions (55). Because of this, anxiety-like behaviors in the EPM and OFT do not always match (56), and the measurement results of different anxiety paradigms are different in different studies. For example, the anxiolytic chlordiazepoxide produces anxiolytic-like effects in the EPM but not in the OFT, whereas the same drug produces anxiolytic-like effects in the OFT but not in the EPM in different animal models (57). In our results, there was also inconsistency between EPM and OFT performance, and we showed that activation of bilateral LHb induced anxiety-like behaviors in the OFT but not in the EPM. Also, inhibition of the release of NKB from the fPAG reversed the alleviating effect of *Tacr3* overexpression in the LHb on pT-ION-related anxiety-like behaviors in the EPM but not in the OFT. The factors that influence behavior mainly include innate genes and acquired environment; in other words, genetics and environment jointly contribute to inter-individual phenotypic variation (58). Thus, another reason for these inconsistencies could be that animals may have different levels of intrinsic anxiety at the time of the EPM vs. the OFT.

Animal tests of anxiety depend on body activity as well as locomotion. In general, in anxious mice motor activity is suppressed (58). It is worth noting that our results showed that chemogenetic activation of LHb neurons reduced the total distance in the OFT. This might be because the LHb is a convenient node in motor suppression. The LHb has inhibitory effects on the downstream dopaminergic and serotonergic systems and thus modulates motor activity (59, 60). However, a previous study reported that optogenetic activation of LHb neurons does not reduce general locomotion in the OFT (3). Our results, along with other reports, suggest that motor control by the LHb might not be generalized, but rather might be specifically linked to different cellular and molecular events.

Different studies have shown contrasting results for the role of NK3R in pain. Specifically, one study showed senktide-mediated nociception in the dorsal PAG (17), while another study showed that micro-infusion of the NK3R agonist into the intra-ventral tegmental area (VTA) or intranucleus accumbens septi caused significant analgesia in the formalin test for tonic pain (61). In our study, we showed that pharmacological inhibition of NK3R in the LHb induced orofacial allodynia and that activating NK3R in the LHb neurons reversed pT-ION-induced allodynia. The contrasting effects of senktide on pain might be related to different localization of NK3R such that senktide in the VTA and LHb causes analgesia while NK3R in the dorsal PAG plays a pain-promoting role. Like the role of NK3R in pain, previous studies have inconsistently reported the anxiolytic-like effects of NK3R (24). Intracerebroventricular injection of senktide significantly increased the frequency of entries and the time spent in the open arms of the EPM, which suggested an anxiolytic action (16). Similar to these reports, we showed that the injection of senktide into the LHb reversed pT-ION-induced anxiety-like behaviors and that pharmacological activation of NK3R in the LHb neurons suppressed abnormal excitation of the LHb neurons. However, another report showed that selective inhibition of NK3R produces anxiolytic and antidepressant-like effects in gerbils (62). This unexpected opposite effect of NK3R on anxiety might in part be due to differences between species. To summarize, these results potentially open a window of opportunity for senktide or similar drugs that activate NK3R to be used for the treatment of chronic pain and comorbid anxiety symptoms.

NK3R has seven hydrophobic transmembrane α-helical regions, which is typical of NK3R’s membership in the family of G-protein–coupled receptors (63, 64). As the natural high-affinity ligand of NK3R, NKB is one of the major tachykinins in mammalian cells (65). NK3R is internalized into endosomes after it is activated by NKB, and its activation is responsible for a wide range of biological actions, including the adenylate cyclase (AC)/cyclic adenosine monophosphate (cAMP)/PKA cascade, the phospholipase C (PLC)/inositol 1,4,5-triphosphate (IP3)/protein kinase C (PKC) cascade, and the Ca^2+^/calmodulin-dependent protein (CaM)/CaMK-II cascade (8). Heterotrimeric G proteins are divided into four families according to the structural homologies of their G α subunits – Gi/o, Gs, Gq/11, and G12/13 – which are responsible for the main properties of the Gαβγ tri-complex (66). Gq/11 mainly activates the PLC/IP3/PKC pathway, and Gs mainly activates the AC/cAMP/PKA pathway, while the G12/13 proteins act as activators to regulate cell shape and motility. However, unlike Gq and Gs, which are stimulatory receptors, the Gi/o proteins are generally described as inhibitory, with AC/PKA and potassium channels as their main effectors (67, 68). In our study, we showed that LHb neuronal excitability was suppressed by activating NK3R, indicating that NK3R belongs to the Gi/o G-protein–coupled receptors and that the possible intracellular mechanisms behind the activation of NK3R might be mediated by the AC/PKA pathway and potassium channels.

In previous work (19) and the present work, we reported that overexpression of NK3R rescued the pT-ION-induced allodynia/anxiety behaviors and the increase in the synaptic activity of LHb neurons, which showed the action of the endogenous NKB ligand. Meanwhile, although NK3R was significantly decreased after pT-ION, we found that the exogenous agonist senktide induced robust suppression of both LHb neuronal firing and allodynia/anxiety behaviors. This is possibly due to the functional adjustability of receptors. Up-regulation and down-regulation of the expression or sensitivity of receptors will occur due to long-term blocking, activation, or other treatment (69, 70). The significant decrease in NK3R expression may lead to the hypersensitization of the remaining NK3R activity; thus, the exogenous agonist senktide induced robust suppression of both LHb neuronal firing and allodynia/anxiety behaviors. Meanwhile, although both NKB and senktide can activate NK3R, the potential and perhaps subtle difference in effects between the endogenous and exogenous ligands may contribute to the observed results.

For slices from pT-ION mice, the NK3R activation decreased the amplitudes but not the frequencies of sEPSCs, indicating potential postsynaptic effects. Neurokinins and glutamate may interact within the solitary tract (71), and glutamate contributes to neurokinin release from neurokinin-containing neurons through the activation of post-synaptic N-methyl-D-aspartate (NMDA) and K-amino-3-hydroxy-5-methyl-4-isoxazole propionic acid (AMPA) receptors. Moreover, the co-expression of NK-3R and NMDA/AMPA receptor subunits was observed in the substantia nigra of basal ganglia nigral neurons, suggesting that NKB/NK3R may be involved in the modulation of neuronal properties and excitotoxicity (72). It has been reported that the activation of NK3R increases the phosphorylation of Thr286-CaMKII in striatal slices (73), and influences phosphorylation levels of AMPA receptors on nigral neurons (74). Therefore, the postsynaptic effects of NK3R activation in pT-ION mice might be due to its modulation of glutamate receptors. In addition, for slices in naive mice, the NK3R inhibition increased the frequencies but not the amplitudes of sEPSCs, indicating potential presynaptic effects. NK3R has been reported to serve as a postsynaptic receptor as well as a presynaptic autoreceptor and heteroreceptor in the globus pallidus of the squirrel monkey (75), and NKB/NK3R signalling stimulates dopamine transmission at the presynaptic site under normal conditions but not in Parkinson’s disease (73). It is possible that blocking the potential presynaptic NK3R may enhance glutamate release from the presynaptic terminals in naive mice.

Maternal deprivation induces LHb hyperactivity through glutamatergic synaptic potentiation that shifts the excitation/inhibition (E/I) balance towards excitation and shows changes in the relative weights of both increased excitatory and decreased inhibitory synaptic inputs onto LHb neurons, and thus produces anxiety-like and depression-like behaviors in adolescent male rats (76). Another study showed that mild traumatic brain injury diminishes spontaneous glutamatergic and GABAergic synaptic activity onto LHb neurons, while the E/I balance is shifted toward excitation through increased suppression of GABAergic transmission (77). In the present study, the GABAergic transmission onto LHb neurons were not recorded. Two recent studies reported that local activation of GAD2^+^ or GABA^+^/GAD65/67^+^ neurons in the LHb suppresses the global activity of the LHb, indicating that there are local inhibitory circuits in the LHb (78, 79). Meanwhile, our preliminary results (data not shown) suggested a decrease in the activity of GABAergic neurons within the LHb after pT-ION, which may contribute to the decrease in the GABAergic transmission onto LHb neurons. Given the increase in glutamatergic transmission found in the present work, we assume that the E/I ratios may be increased, which could explain the changes in LHb neuronal excitability and in firing patterns. However, whether and how the E/I ratios will change after pT-ION and after NK3R intervention needs to be further investigated.

It should be noted that all of the AP properties and excitabilities were measured in the presence of synaptic transmission with typical fast synaptic transmission unblocked. It is possible that some of the observed effects could involve NK3R modulating ion channels on LHb neurons, which could be a contributing factor to the changes in neuronal excitability. It has been reported that NK3R activation selectively prolongs atrial refractoriness by inhibiting background K^+^ channel activity (80). In addition, NK3R activation by the agonist senktide increases Nav1.9 current in enteric neurons, which lowers the action potential threshold and increases the excitability of neurons (81). Whether the intervention of NK3R influences the expression or function of certain ion channels on LHb neurons needs to be further investigated.

Using Tac2-Cre transgenic mice, we first reported NKB^+^ neuronal projections from the fPAG to the LHb. As the “antireward” center, the LHb is reported to receive afferent inputs mainly from the limbic forebrain regions and from the basal ganglia through the stria medullaris fiber tract and to send a major projection to the GABAergic rostromedial tegmental nucleus to suppress dopaminergic neuron activity in the VTA and the substantia nigra pars compacta (82). In previous studies, LHb neurons were reported to have glutamatergic projections to the PAG (35, 83), while no evidence was found that the PAG had any projections to the LHb. Here, our results show NKB+ neuronal projections from the fPAG to the LHb and provide evidence that mutual projections exist between the LHb and the fPAG. Indeed, the LHb not only has a mutual projection with the fPAG, and it also has projections with the median raphe nucleus, diagonal band nuclei, VTA, and so on (82). For example, the medial part of the LHb receives both glutamatergic projections and GABAergic projections from the limbic areas, including the VTA, and in return it outputs predominantly glutamatergic projections to the neurons of aminergic nuclei, including dopaminergic and GABAergic neurons in the VTA (84, 85). In the PAG, both glutamatergic and GABAergic-releasing output neurons have been described (86). While the majority of GABAergic neurons in the PAG directly or indirectly regulate activity in efferent pathways and are presumed to be inhibitory interneurons (87), the output projection neurons in the PAG are predominantly glutamatergic neurons (26, 27). Based on these anatomical projections, we had reasons to test if fPAG^NKB^ → LHb would affect the role of NK3R in the LHb. As we show in this study, chemogenetic inhibition of the release of NKB in the fPAG reversed the alleviating effect of *Tacr3* overexpression on pT-ION-induced allodynia and anxiety-like behaviors, indicating that fPAG^NKB^ → LHb regulates orofacial allodynia and anxiety-like behaviors in pT-ION mice. Compared with the NKB^–^ neurons, the firing rate of NKB^+^ gluatamatergic neurons was significantly higher after pT-ION, and the rheobase was significantly lower, which suggests a more active status of NKB^+^ gluatamatergic neurons. This may be the compensatory increase in ligand due to the decrease in NK3R in the LHb after pT-ION.

However, the current study only investigated the function of NK3R in the LHb. Whether NK3R in other brain regions mediates the pain and anxiety comorbidity is unclear based on the current data. Moreover, all of the experiments were performed in rodents, and therefore the results should be tested and confirmed in human patients suffering from chronic pain and anxiety comorbidity.

## 5 Conclusion

**Figure 11:**
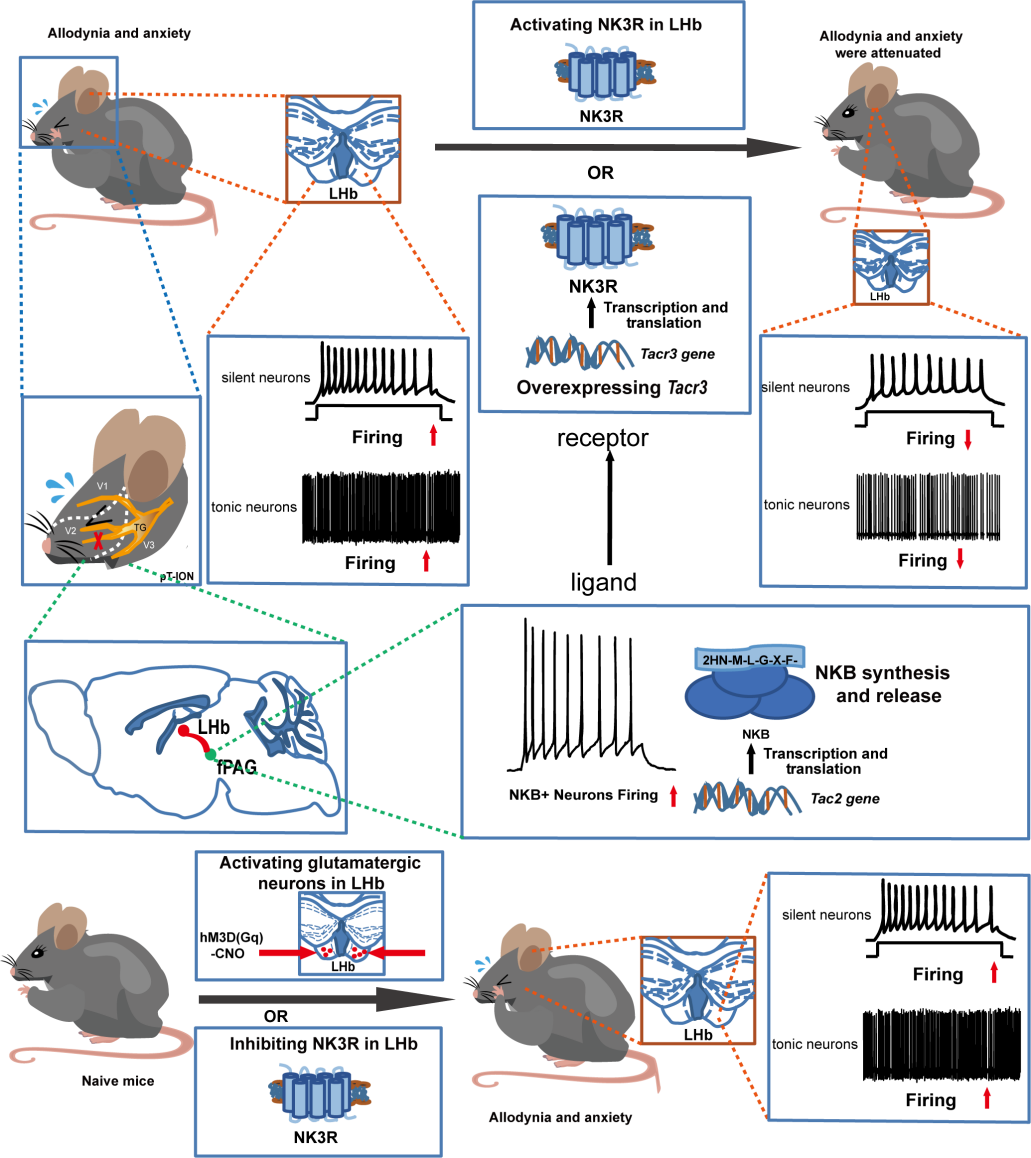
Schematic diagram of the role of NK3R in the LHb in the development of orofacial allodynia and comorbid anxiety. Under normal physiological conditions, NK3R in the LHb is protective. Trigeminal nerve injury leads to the downregulation of NK3R, and thus the orofacial allodynia and anxiety-like behaviors are induced due to the hyperactivity of LHb neurons. Its ligand NKB is mainly from the fPAG, and fPAG ^NKB^ → LHb projections regulate orofacial allodynia and comorbid anxiety.

In summary, we have found that NK3R in the LHb mediates orofacial allodynia and pain-related anxiety. Neurons in the LHb are hyperactive in trigeminal neuralgia combined with anxiety, and activating NK3R in the LHb suppresses the abnormal excitation and has analgesic and anxiolytic effects. In addition, the previously unrecognized fPAG ^NKB^ → LHb circuit is involved in pain and anxiety comorbidity (**Figure 11**). Furthermore, the male and female Tac2-cre mice behaved similarly in the comparison of both painful and anxiety-like behaviors. We propose that NK3R-targeted interventions might be worth pursuing for treating pain and pain-related anxiety in trigeminal neuralgia.

## Data availability statement

The data that support the findings of this study are available from the corresponding author upon reasonable request.

## Ethics statement

The animal study was reviewed and approved by the Institutional Animal Care and Use Committee of Fudan University (20160225-074).

## Author contributions

Y-XC, Y-QiW, and W-WZ designed the experiments. Y-XC and W-WZ wrote the paper. W-WZ, TC, and S-YL carried out most of the experiments. X-YW, W-BL, and Y-QuW participated in some of the behavioral experiments and data analysis. Q-LM-Y, and W-LM provided timely advice on the research.
