# Supplementary material for: Tachykinin receptor 3 in the lateral habenula alleviates pain and anxiety comorbidity in mice
Source: Front Immunol. 2023 Jan 23;14:1049739. doi: 10.3389/fimmu.2023.1049739 (PMC9900122; doi:10.3389/fimmu.2023.1049739)
Supplement: Supplementary file 1 [file DataSheet_1.docx]

Supplementary Information for

**Tachykinin receptor 3 in the lateral habenula alleviates pain and anxiety comorbidity in mice**

Figure and table legends


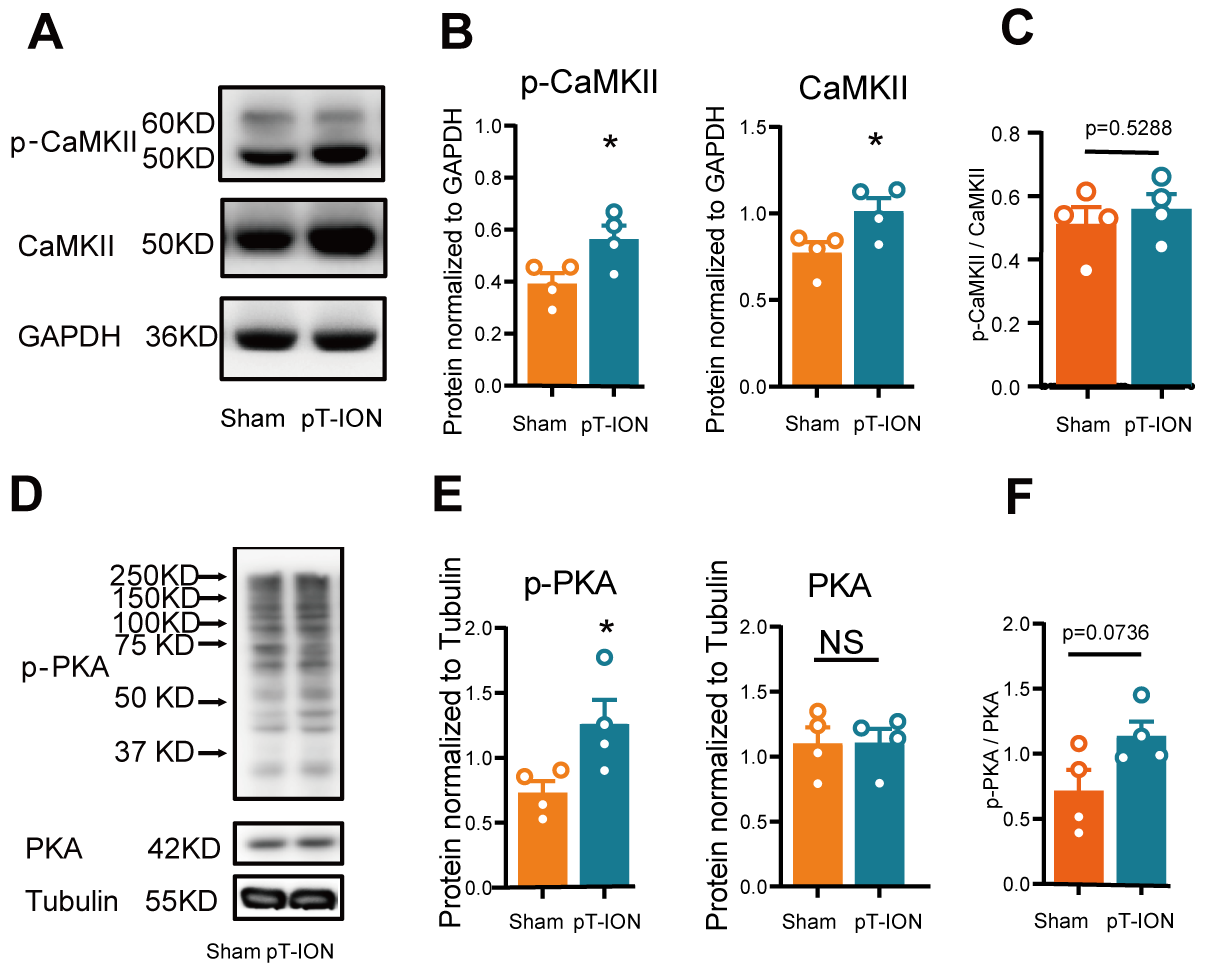


**Fig. S1 Neuronal activation-associated protein expression in the LHb.**

(A-C) The protein expression of p-CaMKII and CaMKII in the LHb was increased in pT-ION mice while the p-CaMKII/CaMKII ratio was unchanged. * p < 0.05 vs. sham control. (D-F) The expression of PKA was unchanged, while the p-PKA level and the p-PKA/PKA ratio in the LHb were increased in pT-ION mice. *p < 0.05 vs. sham control.

**
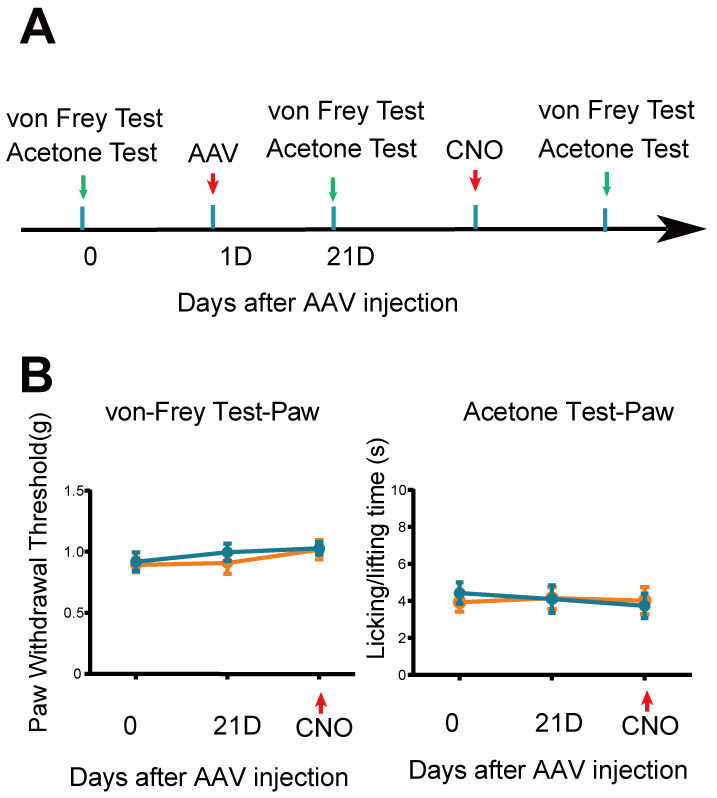
**

**Fig. S2 The effect of chemogenetic activation of bilateral LHb neurons on allodynia in the paw.**

1. The time schedule of behavioral testing, virus injection, and CNO administration. (B) Mechanical allodynia (left) and cold allodynia (right) of the paw were unchanged by AAV-CaMKIIa-hM3D(Gq)-mCherry-WPREs-pA virus injection into the bilateral LHb and subsequent CNO exposure.


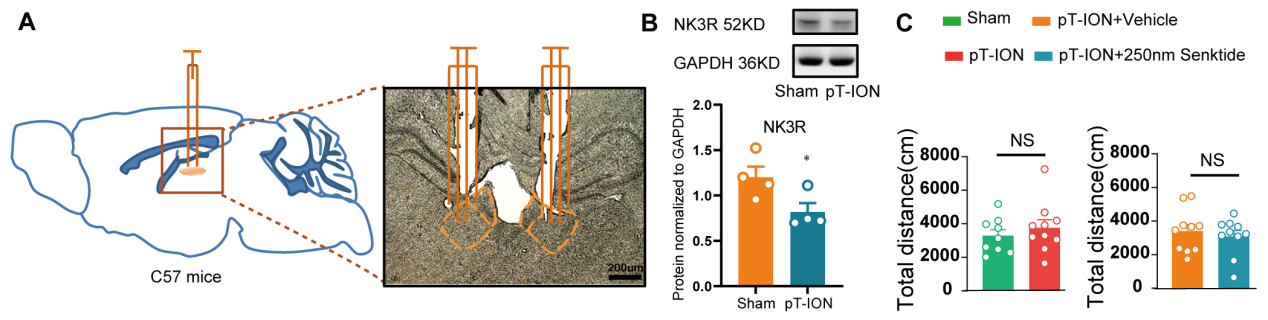


**Fig. S3 The expression of NK3R and the effect of NK3R activation on the total distance in the OFT.**

(A) Schematic (left) and typical image (right) of the bilateral implantation of the cannulae in the LHb. (B) The expression of NK3R in the LHb was decreased after pT-ION. *p < 0.05 vs. sham. (C) The total distance remained unchanged on day 21 after pT-ION and after bilateral senktide injection into the LHb (n = 10 mice per group).


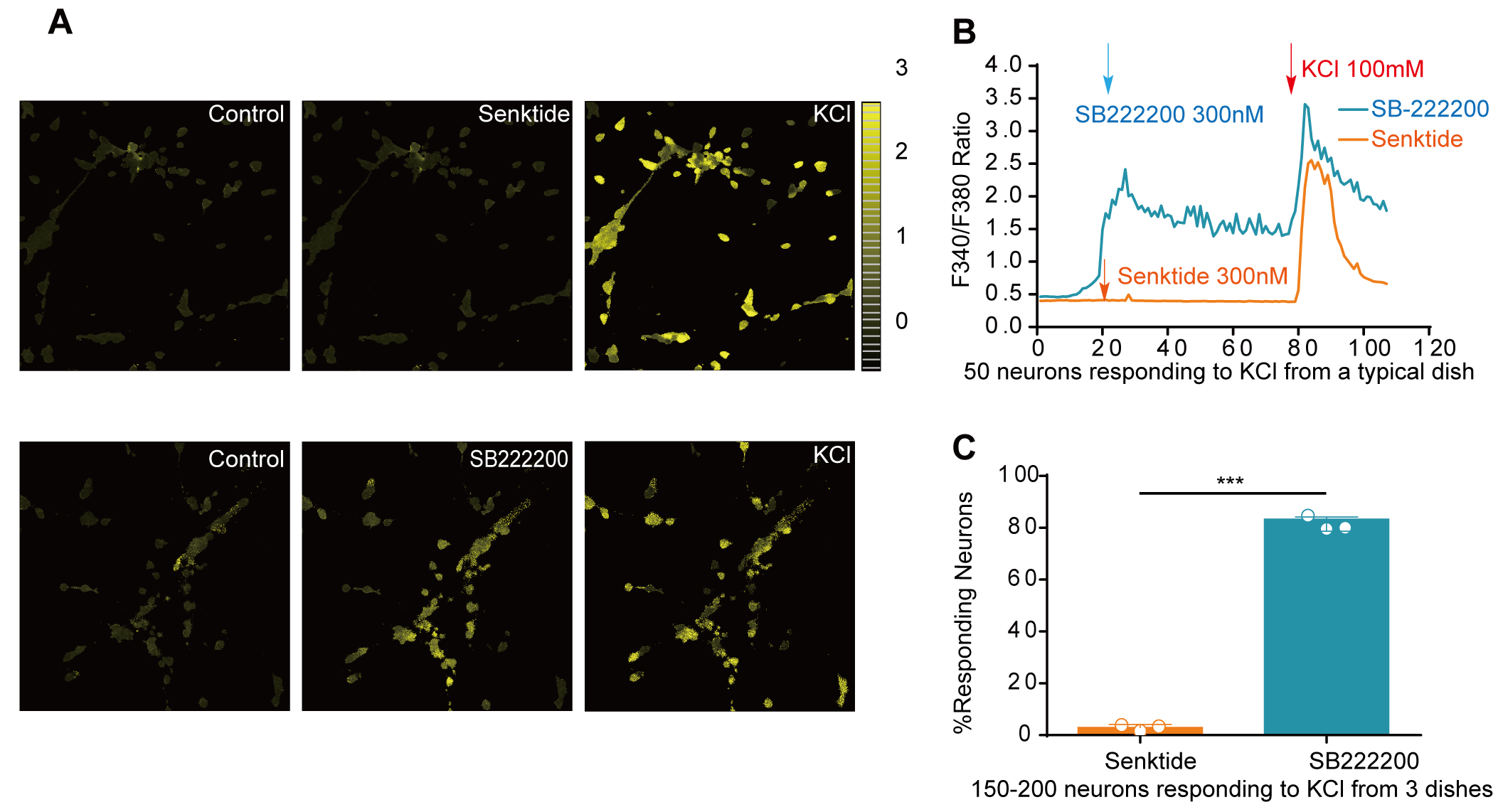


**Fig. S4 Neurons were activated by the NK3R antagonist SB222200.**

1. B) Typical images (A) and plots (B) of Ca^2+^ transients in primary hippocampal neurons with SB222200 or senktide. The colored bar indicates the degree of calcium influx. (C) Data for responding neurons with senktide and SB222200 (***p < 0.001 vs. senktide, n = 150–200 neurons responding to KCl from three separate culture dishes).


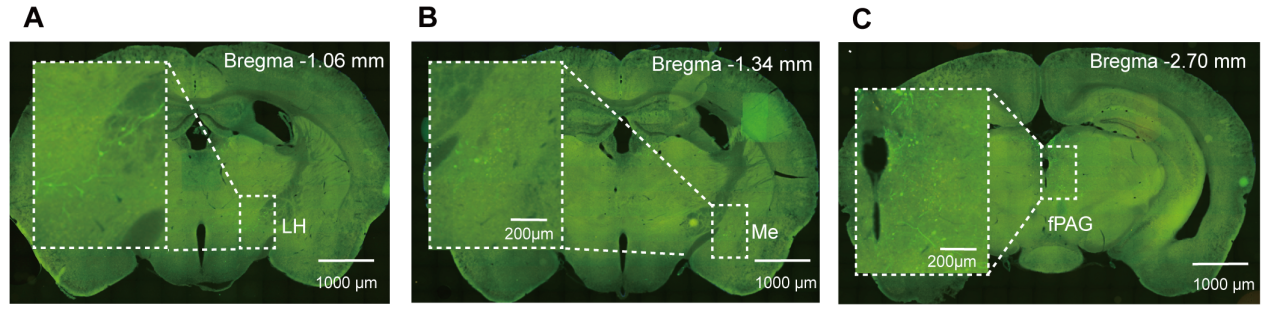


**Fig. S5 Retrograde fluorescence expression in the brain after injection of Retro-rAAV-EF1a-DIO–EGFP into the LHb of Tac2-Cre mice.**

1. C) Representative images of the lateral hypothalamic area (A), medial amygdaloid nucleus (B), and fPAG (C) showing EGFP+ neurons in a Tac2-Cre mouse with LHb injection of Retro-rAAV-EF1a-DIO–EGFP.


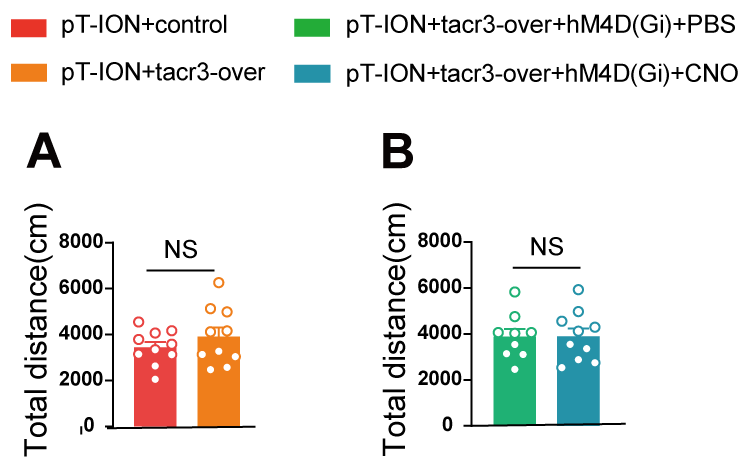


**Fig. S6 Total distance in the OFT related to Fig 10.**

(A) The total distances were unchanged after Tacr3 overexpression (n = 10 mice per group). (B) The total distances remained unchanged after chemogenetic inhibition of fPAG^NKB^ projections in mice subject to Tacr3 overexpression (n = 10 mice per group).


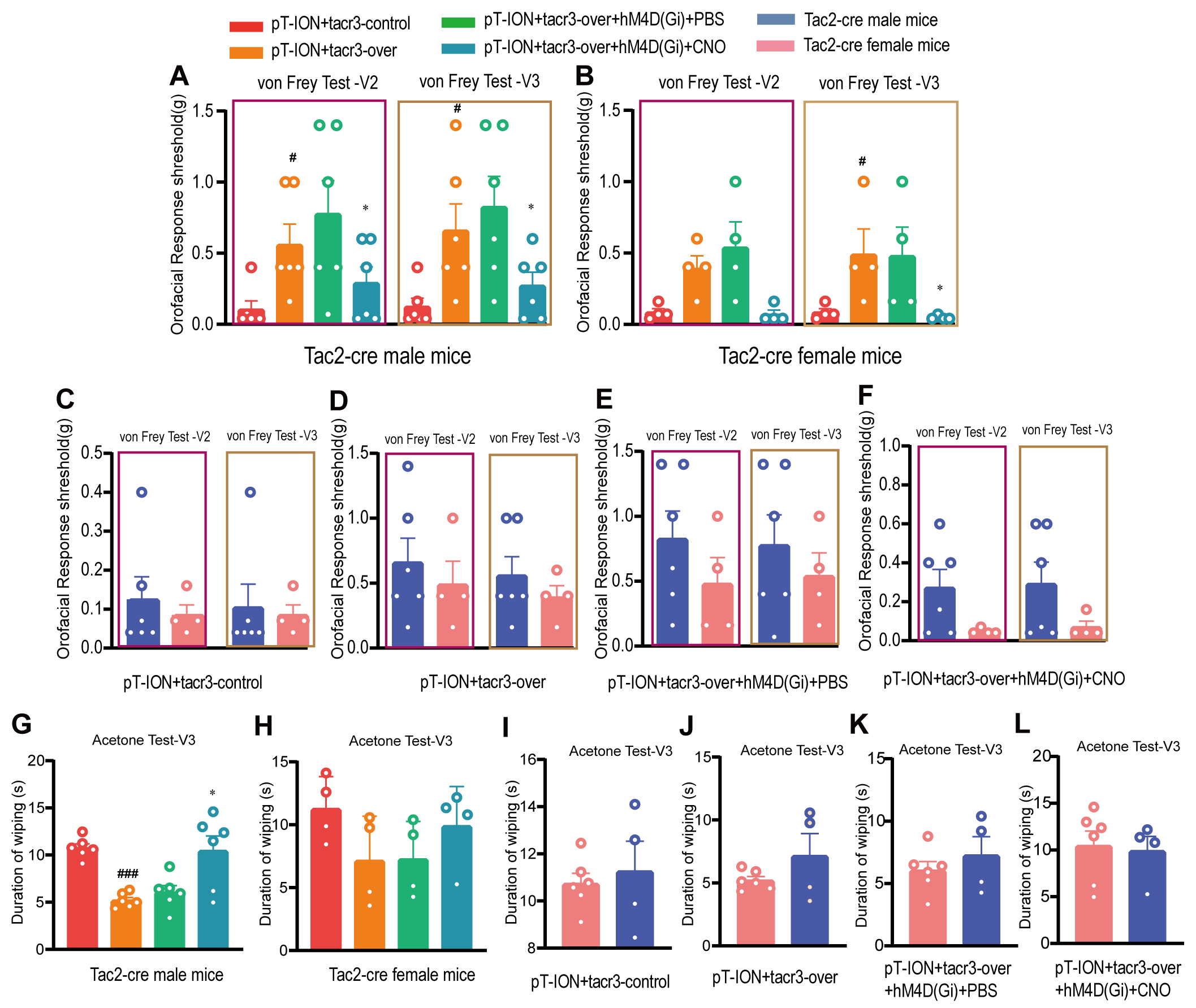


**Fig. S7 Tac2-Cre male and female mice behaved similarly in terms of orofacial pain behaviors.**

1. B) Both Tac2-Cre male (A) and female mice (B) showed that pT-ION-induced V2 allodynia and V3 allodynia were improved by bilateral Tacr3 overexpression in the LHb (#p < 0.05 vs. the pT-ION+tacr3-control group, n = 4 mice in the female group, n = 6 mice in the male group). The V3 allodynia was reversed by bilateral inhibition of fPAG^NKB^ projections to the LHb, while the V2 allodynia only showed a decreasing trend (*p < 0.05 vs. the corresponding control group). (C-F) The pT-ION+tacr3-control group (C), the pT-ION+tacr3-overexpression group (D), the pT-ION+tacr3-over+hM4D(Gi)+PBS group (E), and the pT-ION+tacr3-over+hM4D(Gi)+CNO group (F) all showed no difference in pT-ION-induced V2 allodynia and V3 allodynia between Tac2-Cre male and female mice. (G-H) pT-ION-induced V3 cold allodynia was improved by Tacr3 overexpression, which was reversed by chemogenetic inhibition of fPAG^NKB^ projections to the LHb in the Tac2-Cre male mice (G), while this only showed a decreasing trend in Tac2-Cre female mice (H) (###p < 0.001 vs. the pT-ION+tacr3-control group, *p < 0.05 vs. the pT-ION+tacr3-over+hM4D(Gi)+PBS group). (I-L) The pT-ION+tacr3-control group (I), the pT-ION+tacr3-overexpression group (J), the pT-ION+tacr3-over+hM4D(Gi)+PBS group (K), and the pT-ION+tacr3-over+hM4D(Gi)+CNO group (L) all showed no difference in pT-ION-induced V3 cold allodynia between Tac2-Cre male and female mice.


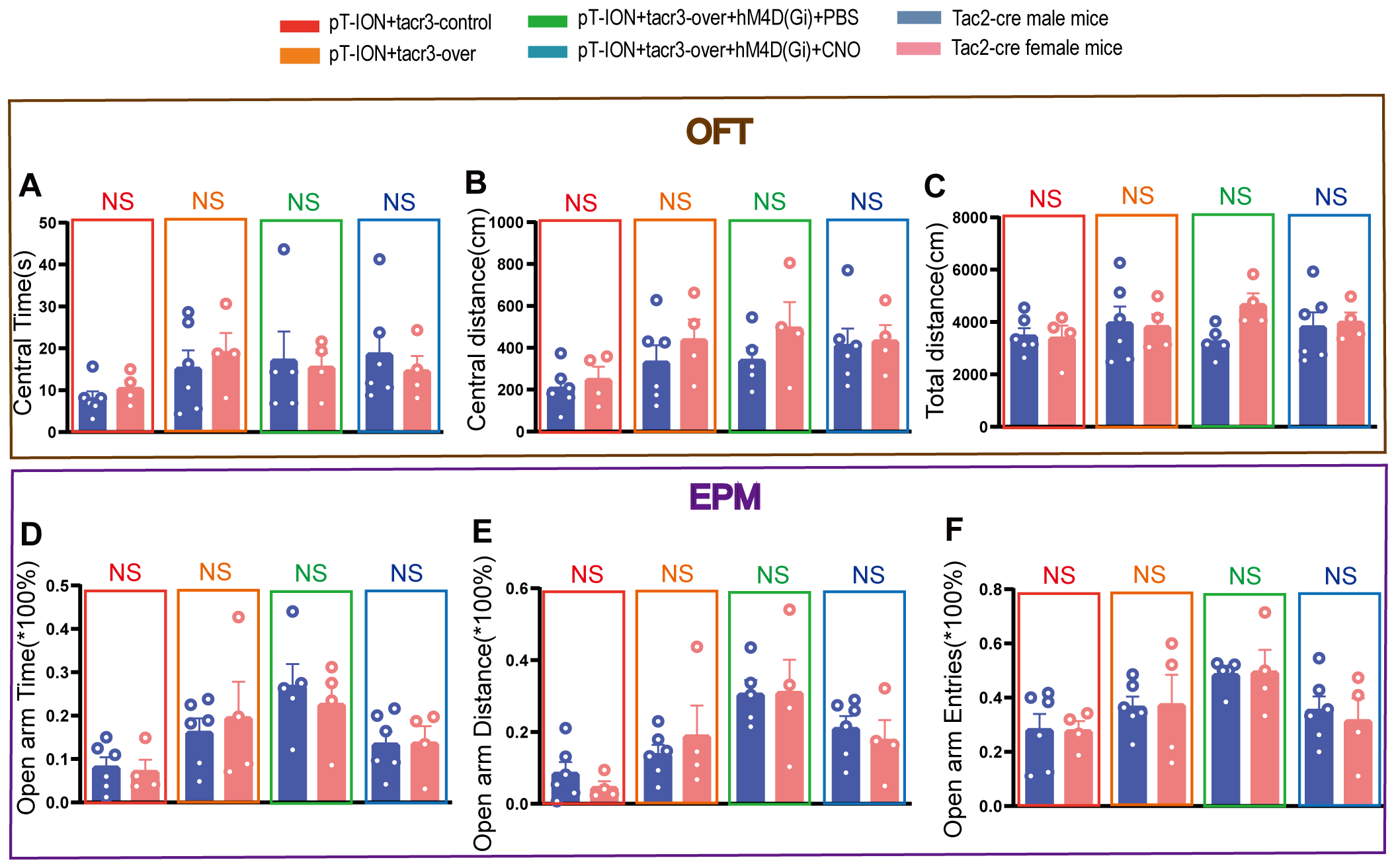


**Fig. S8 Tac2-Cre male and female mice showed similar anxiety-like behaviors.**

(A-C) No difference was seen in the central time (A), central distance (B), or total distance (C) in the OFT between Tac2-Cre male and female mice. (D-F) No difference was seen in the percentage of time (D), distance (E), and entries (F) in the open-arm of the EPM between Tac2-Cre male and female mice (n = 4 mice in the female group, n = 6 mice in the male group).

**Table S1 Key resources table**

| **Reagent or resource** | **Source** | **Identifier** |
| --- | --- | --- |
| **Antibodies** |  |  |
| anti-Glutamate | Sigma | G6642 |
| anti-GABA; | Sigma | A2052 |
| anti-CamKII-α | CST | 50049 |
| anti-p-CamKII | CST | 12716 |
| anti-PKA C-α | CST | 4782 |
| anti-p-PKA | CST | 9624 |
| anti-NK3R | NOVUS | NB300-102 |
| **Virus strains** |  |  |
| rAAV-CaMKIIa-hM3D(Gq)-mCherry-WPREs-pA | BrainVTA | PT-0049 |
| Retro-rAAV-EF1a-DIO–EGFP- WPRE-pA 2/R | BrainVTA | PT-0795 |
| rAAV-EF1a-DIO–mCherry-hGH | BrainVTA | PT-0013 |
| rAAV-hSyn-CRE-EGFP-WPRE-hGH | BrainVTA | PT-1168 |
| rAAV-EF1α-DIO-EGFP-WPRE-hGH | BrainVTA | PT-0795 |
| rAAV-EF1α-fDIO-EGFP-WPRE-hGH | BrainVTA | PT-1047 |
| rAAV-EF1α-DIO-FLP-WPRE-hGH | BrainVTA | PT-0075 |
| pAAV-CMV-EGFP-2A-Tacr3-3FLAG | OBIO Technology | H12378 |
| rAAV-EF1α-DIO-hCHR2(H134R) -mCherry-WPRE | BrainVTA | PT-0002 |
| rAAV-CaMKIIa-DIO-hM4D(Gi)-mCherry-WPRE-hGH | BrainVTA | PT-1143 |
| **Chemicals, peptides, and recombinant proteins** |  |  |
| Senktide | MedChemExpress | HY-P0187 |
| SB-222200 | MedChemExpress | HY-15722 |
| clozapine-N-oxide (CNO) | Sigma | C0832 |
| **Experimental models** |  |  |
| C57BL/6J | Experimental Animal Center of the Chinese Academy of Sciences | N/A |
| **Software and algorithms** |  |  |
| GraphPad Prism 8 | GraphPad Software | https://www.graphpad.com |
| Clampfit | Molecular Devices | https://www.moleculardevices.com/ |
| ImageJ | Charles River Laboratories | N/A |
| Adobe Illustrator CS5 | Adobe APE | Adobe Linguistic Library 5.0.0 |
| FV10.ASW Version.4.2b | ComponentOne LLC. | http://www.grapecity.com/ |
| **Other** |  |  |
| 4′,6-diamidino-2-phenylindole (DAPI) Fluoromount-G® | Southern Biotech | 0100–20 |
| Cannulae | RWD | N/A |

**Table S2 Statistical analysis results for one-way ANOVA**

| Figure | Panel | Factors | F | *p* | DFn | DFd |
| --- | --- | --- | --- | --- | --- | --- |
| 4 | B | Numbers of spikes | 6.554 | 0.0078 | 2 | 17 |
| 4 | C | Rheobase | 26.4 | <0.0001 | 2 | 17 |
| 4 | D | Threshold | 0.9856 | 0.3873 | 2 | 25 |
| 6 | B | Numbers of spikes | 4.758 | 0.0177 | 2 | 25 |
| 6 | C | Rheobase | 8.427 | 0.0016 | 2 | 25 |
| 6 | D | Threshold | 0.0758 | 0.9272 | 2 | 25 |

**Table S3 Statistical analysis results for two-way RM ANOVA**

| Figure | Panel | Interaction  DFn,  DFd F *p* | | | Time  DFn,  DFd F *p* | | | Treatment  DFn,  DFd F *p* | | |
| --- | --- | --- | --- | --- | --- | --- | --- | --- | --- | --- |
| 1 | K | 10,210 | 15.93 | <0.0001 | 2.9,61.4 | 457.6 | <0.0001 | 1,21 | 19.66 | 0.0002 |
| 2 | C | 2,42 | 1.909 | 0.1609 | 2,42 | 3.492 | 0.0395 | 1,42 | 2.705 | 0.1075 |
| 2 | D | 2,56 | 1.811 | 0.1730 | 1.85,51.9 | 1.591 | 0.2146 | 1.28 | 3.875 | 0.0590 |
| 2 | E | 2,56 | 0.4293 | 0.6531 | 1.8,50.8 | 1.012 | 0.3643 | 1,28 | 0.0024 | 0.9613 |
| S2 | C.von-Frey | 2,28 | 0.2049 | 0.8160 | 2,28 | 1.735 | 0.1948 | 1,14 | 0.3360 | 0.5714 |
| S2 | C.Acetone | 2,28 | 0.2396 | 0.7885 | 2,28 | 0.1522 | 0.8595 | 1,14 | 0.0085 | 0.9277 |
| 3 | C. Senktide | 2,36 | 4.004 | 0.0268 | 1.4,25.3 | 31.59 | <0.0001 | 1,18 | 6.839 | 0.0175 |
| 3 | D. Senktide | 2,36 | 4.004 | 0.0268 | 1.4,25.3 | 31.59 | <0.0001 | 1,18 | 6.839 | 0.0175 |
| 3 | E. Senktide | 1,18 | 0.7791 | 0.3891 | 1,18 | 2.145 | 0.1602 | 1,18 | 4.565 | 0.0466 |
| 5 | C | 2,36 | 6.66 | 0.0035 | 2,36 | 4.49 | 0.0182 | 1,18 | 9.62 | 0.0062 |
| 5 | D | 2,36 | 6.418 | 0.0041 | 1.58,28.4 | 5.256 | 0.0167 | 1,18 | 10.68 | 0.0043 |
| 5 | E | 1,18 | 12.46 | 0.0024 | 1,18 | 5.065 | 0.0372 | 1,18 | 5.184 | 0.0352 |
| 9 | B | 9,198 | 1.202 | 0.2955 | 9,198 | 95.26 | <0.0001 | 1,21 | 19.66 | 0.0002 |
| 10 | E | 3,36 | 1.893 | 0.1482 | 3,36 | 5.580 | 0.0030 | 1,36 | 2.193 | 0.1474 |
| 10 | F | 3,72 | 2.037 | 0.1163 | 3,72 | 11.55 | <0.0001 | 1,72 | 3.447 | 0.0675 |
| 10 | G | 3,72 | 5.4441 | 0.002 | 3,72 | 12.93 | <0.0001 | 1,72 | 14.02 | 0.0004 |
| 10 | H | 4,90 | 1.852 | 0.1258 | 4,90 | 2.809 | 0.0301 | 1,90 | 0.4486 | 0.5047 |
| 10 | I | 4,90 | 1.956 | 0.1080 | 4,90 | 8.371 | <0.0001 | 1,90 | 2.077 | 0.1530 |
| 10 | J | 3,72 | 2.021 | 0.1186 | 3,72 | 5.017 | 0.0032 | 1,72 | 3.334 | 0.0720 |

**Table S4 Statistical analysis results for Student’s t-test**

| Fig Panel | | | Comparation | Student’s T-test | | | | T | | *p* | DFn,DEd | | | | |  | Fig Panel Comparation Student’s T-test | | | | | T | | p | DFn,DEd | |  |
| --- | --- | --- | --- | --- | --- | --- | --- | --- | --- | --- | --- | --- | --- | --- | --- | --- | --- | --- | --- | --- | --- | --- | --- | --- | --- | --- | --- |
| S1 | B | | p-CaMKⅡ | | Unpaired | | 1.735 | | 0.0396 | | | | 3,3 | |  | | 4 | I | Amplitude | Paired | 3．670 | | 0.0278 | | | -,- | |
| S1 | B | | CaMKⅡ | | Unpaired | | 1.580 | | 0.0464 | | | | 3,3 | |  | | 4 | J | Frequency | Paired | 1.918 | | 0.1036 | | | -,- | |
| S1 | E | | p-PKA | | Unpaired | | 4.364 | | 0.0429 | | | | 3,3 | |  | | 5 | G | Open-arm time | Unpaired | 1.292 | | 0.003 | | | 9,9 | |
| S1 | E | | PKA | | Unpaired | | 1.308 | | 0.9793 | | | | 3,3 | |  | | 5 | H | Open-arm distance | Unpaired | 1.146 | | 0.0445 | | | 9,9 | |
| S1 | C | | p-CaMKⅡ/ CaMKⅡ | | Unpaired | | 0.6683 | | 0.5288 | | | | 3,3 | |  | | 5 | I | Open-arm entries | Unpaired | 1.316 | | 0.5991 | | | 9,9 | |
| S1 | F | | p-PKA/PKA | | Unpaired | | 2.164 | | 0.0736 | | | | 3,3 | |  | | 5 | K | Central time | Unpaired | 3.168 | | 0.0019 | | | 9,9 | |
| 1 | D | | Neuronal RMP | | Unpaired | 1.824 | | | 0.0373 | | | 27,34 | | |  | | 5 | L | Central distance | Unpaired | 1.443 | | 0.0227 | | | 9,9 | |
| 1 | E | | silent | | Unpaired | 1.148 | | | 0.2565 | | | 19,31 | | |  | | 5 | M | Total distance | Unpaired | 1.815 | | 0.8145 | | | 9,9 | |
| 1 | E | | Tonic-firing | | Unpaired | 1.324 | | | 0.1947 | | | 18,15 | | |  | | 6 | F | Spontaneous spikes | Paired | 7.115 | | 0.0021 | | | -,- | |
| 1 | E | | Burst-firing | | Unpaired | 0.3111 | | | 0.776 | | | -,- | | |  | | 6 | G | Threshold | Unpaired | 0.5340 | | 0.6125 | | | 3,3 | |
| 1 | H | | Frequency | | Unpaired | 2.327 | | | 0.0355 | | | 7,7 | | |  | | 6 | I | Amplitude | Paired | 1.694 | | 0.1340 | | | -,- | |
| 1 | I | | Threshold | | Unpaired | 0 | | | >0.999 | | | 7,7 | | |  | | 6 | J | Frequency | Paired | 2.599 | | 0.0355 | | | -,- | |
| 1 | L | | Rheobase | | Unpaired | 2.890 | | | 0.0088 | | | 12,9 | | |  | | 7 | K | Cells | Unpaired | 1.303 | | 0.6553 | | | 6,6 | |
| 1 | M | | Threshold | | Unpaired | 1.101 | | | 0.2833 | | | 9,12 | | |  | | 7 | N | Cells | Unpaired | 4.284 | | <0.0001 | | | 6,6 | |
| 2 | G | | Open-arm time | | Unpaired | 2.284 | | | 0.6144 | | | 14,14 | | |  | | 8 | C | Amplitude | Paired | 8.853 | | 0.0009 | | | -,- | |
| 2 | H | | OFT distance | | Unpaired | 1.139 | | | 0.6757 | | | 14,14 | | |  | | 8 | D | Frequency | Paired | 3.416 | | 0.0269 | | | -,- | |
| 2 | I | | Open-arm entries | | Unpaired | 1.569 | | | 0.1063 | | | 14,14 | | |  | | 9 | D | Spike number | Unpaired | 2.117 | | 0.0458 | | | 11,11 | |
| 2 | K | | Central time | | Unpaired | 1.869 | | | 0.036 | | | 12,11 | | |  | | 9 | E | RMP | Unpaired | 0.2793 | | 0.7826 | | | 11,11 | |
| 2 | L | | Central distance | | Unpaired | 1.582 | | | 0.0261 | | | 12,11 | | |  | | 9 | F | Rheobase | Unpaired | 2.173 | | 0.0408 | | | 11,11 | |
| 2 | M | | Central/Total distance | | Unpaired | 1.501 | | | 0.0496 | | | 12,11 | | |  | | 9 | G | Threshold | Unpaired | 1.220 | | 0.2353 | | | 11,11 | |
| 3 | C | | pT-ION vs. sham 21D | | Unpaired | 2.439 | | | 0.0312 | | | 9,10 | | |  | | 10 | L | Open-arm distance | Unpaired | 2.195 | | 0.0415 | | | 9,9 | |
| 3 | D | | pT-ION vs. sham 21D | | Unpaired | 7.160 | | | 0.002 | | | 9,10 | | |  | | 10 | L | Open-arm time | Unpaired | 2.517 | | 0.0215 | | | 9,9 | |
| 3 | E | | pT-ION vs. sham 21D | | Unpaired | 3.036 | | | 0.0007 | | | 10,9 | | |  | | 10 | L | Open-arm entries | Unpaired | 1.542 | | 0.1405 | | | 9,9 | |
| 3 | G_1_ | | Open-arm time | | Unpaired | 1.169 | | | 0.0317 | | | 8,8 | | |  | | 10 | N | Central distance | Unpaired | 2.194 | | 0.0416 | | | 9,9 | |
| 3 | G_2_ | | OFT distance | | Unpaired | 1.871 | | | 0.0228 | | | 8,8 | | |  | | 10 | N | Central time | Unpaired | 2.380 | | 0.0286 | | | 9,9 | |
| 3 | G_3_ | | Open-arm entries | | Unpaired | 1.029 | | | 0.0435 | | | 8,8 | | |  | | 10 | R | Central distance | Unpaired | 0.1377 | | 0.8921 | | | 8,9 | |
| 3 | K_1_ | | Central time | | Unpaired | 4.396 | | | 0.0244 | | | 8,9 | | |  | | 10 | P | Open-arm distance | Unpaired | 2.211 | | 0.0410 | | | 8,9 | |
| 3 | K_2_ | | Central distance | | Unpaired | 2.019 | | | 0.1741 | | | 8,9 | | |  | | 10 | P | Open-arm time | Unpaired | 2.867 | | 0.0107 | | | 8,,9 | |
| 3 | I_1_ | | Open-arm time | | Unpaired | 2.021 | | | 0.0125 | | | | | 9,9 |  | | 10 | P | Open-arm entries | Unpaired | 2.728 | | 0.0143 | | | 8,9 | |
| 3 | I_2_ | | OFT distance | | Unpaired | 3.153 | | | 0.0055 | | | | 9,9 | |  | | 10 | R | Central time | Unpaired | 0.1243 | | 0.9023 | | | 8,9 | |
| 3 | I_3_ | | Open-arm entries | | Unpaired | 1.209 | | | 0.0028 | | | | 9,9 | |  | | S_6_ | A | Total distance | Unpaired | 1.026 | | 0.3184 | | | 9,9 | |
| 3 | M_1_ | | Central time | | Unpaired | 5.454 | | | 0.002 | | | | 9,9 | |  | | S_6_ | B | Total distance | Unpaired | 0.0151 | | 0.9881 | | | 9,9 | |
| 3 | M_2_ | | Central distance | | Unpaired | 3.670 | | | 0.0068 | | | | 9,9 | |  | | S_7_ | A | V2-Tacr3-over | Unpaired | 2.728 | | 0.0213 | | | 5,5 | |
| S_2_ | D | | Total distance | | Unpaired | 1.235 | | | 0.0299 | | | | 12,11 | |  | | S_7_ | A | V2-hM4D(Gi)-CNO | Unpaired | 2.377 | | 0.0388 | | | 5,5 | |
| S_3_ | B | | NK3R | | Unpaired | 1.464 | | | 0.0477 | | | | 3,3 | |  | | S_7_ | A | V3-Tacr3-over | Unpaired | 2.919 | | 0.0153 | | | 5,5 | |
| S_3_ | C_1_ | | Total distance | | Unpaired | 2.047 | | | 0.4406 | | | | 8,9 | |  | | S_7_ | A | V3-hM4D(Gi)-CNO | Unpaired | 1.8891 | | 0.0880 | | | 5,5 | |
| S_3_ | C_2_ | | Total distance | | Unpaired | 1.322 | | | 0.5639 | | | | 9,9 | |  | | S_7_ | B | V2-Tacr3-over | Unpaired | 2.237 | | 0.0666 | | | 3,3 | |
| S_4_ | C | | Responding neurons | | Unpaired | 6.043 | | | <0.0001 | | | | 2,2 | |  | | S_7_ | B | V2-hM4D(Gi)-CNO | Unpaired | 2.140 | | 0.0762 | | | 3,3 | |
| 4 | F | | Spontaneous spikes | | Paired | 3.248 | | | 0.0476 | | | -,- | | |  | | S_7_ | B | V3-Tacr3-over | Unpaired | 3.256 | | 0.0173 | | | 3,3 | |
| 4 | G | Threshold | | | Unpaired | 0.1796 | | | 0.8634 | | | | 3,3 | |  | | S_7_ | B | V3-hM4D(Gi)-CNO | Unpaired | 2.607 | | 0.0403 | | | 3,3 | |
| S_8_ | B | Tacr3-control | | | Unpaired | 0.6109 | | | 0.5582 | | | | 3,5 | |  | | S_7_ | C | V2 | Unpaired | 0.5239 | | 0.6110 | | | 5,3 | |
| S_8_ | B | Tacr3-over | | | Unpaired | 0.8523 | | | 0.4188 | | | | 5,3 | |  | | S_7_ | C | V3 | Unpaired | 0.2604 | | 0.8011 | | | 5,3 | |
| S_8_ | B | hM4D(Gi)-PBS | | | Unpaired | 1.210 | | | 0.2656 | | | | 3,4 | |  | | S_7_ | D | V2 | Unpaired | 1.104 | | 0.3018 | | | 5,3 | |
| S_8_ | B | hM4D(Gi)-CNO | | | Unpaired | 0.1844 | | | 0.8583 | | | | 5,3 | |  | | S_7_ | D | V3 | Unpaired | 0.3053 | | 0.7609 | | | 5,3 | |
| S_8_ | C | Tacr3-control | | | Unpaired | 0.1563 | | | 0.8795 | | | | 3,5 | |  | | S_7_ | E | V2 | Unpaired | 1.115 | | 0.2971 | | | 5,3 | |
| S_8_ | C | Tacr3-over | | | Unpaired | 0.1660 | | | 0.8723 | | | | 5,3 | |  | | S_7_ | E | V3 | Unpaired | 0.7404 | | 0.4802 | | | 5,3 | |
| S_8_ | C | hM4D(Gi)-PBS | | | Unpaired | 3.026 | | | 0.0192 | | | | 3,4 | |  | | S_7_ | F | V2 | Unpaired | 1.936 | | 0.0889 | | | 5,3 | |
| S_8_ | C | hM4D(Gi)-CNO | | | Unpaired | 0.2375 | | | 0.8183 | | | | 5,3 | |  | | S_7_ | F | V3 | Unpaired | 1.558 | | 0.1578 | | | 5,3 | |
| S_8_ | D | Tacr3-control | | | Unpaired | 0.2960 | | | 0.7748 | | | | 5,3 | |  | | S_7_ | G | V2-Tacr3-over | Unpaired | 10.09 | | <0.0001 | | | 5,5 | |
| S_8_ | D | Tacr3-over | | | Unpaired | 0.4482 | | | 0.6659 | | | | 3,5 | |  | | S_7_ | G | V2-hM4D(Gi)-CNO | Unpaired | 2.510 | | 0.0309 | | | 5,5 | |
| S_8_ | D | hM4D(Gi)-PBS | | | Unpaired | 0.5655 | | | 0.5894 | | | | 4,3 | |  | | S_7_ | H | V3-Tacr3-over | Unpaired | 1.883 | | 0.1087 | | | 3,3 | |
| S_8_ | D | hM4D(Gi)-CNO | | | Unpaired | 0.0495 | | | 0.9617 | | | | 3,5 | |  | | S_7_ | H | V3-hM4D(Gi)-CNO | Unpaired | 1.218 | | 0.2688 | | | 3,3 | |
| S_8_ | E | Tacr3-control | | | Unpaired | 0.9727 | | | 0.3592 | | | | 5,3 | |  | | S_7_ | I | V2-Tacr3-over | Unpaired | 0.4550 | | 0.6612 | | | 3,5 | |
| S_8_ | E | Tacr3-over | | | Unpaired | 0.7 | | | 0.5038 | | | | 3,5 | |  | | S_7_ | J | V2-hM4D(Gi)-CNO | Unpaired | 1.342 | | 0.2163 | | | 3,5 | |
| S_8_ | E | hM4D(Gi)-PBS | | | Unpaired | 0.0512 | | | 0.9606 | | | | 3,4 | |  | | S_7_ | K | V3-Tacr3-over | Unpaired | 0.8135 | | 0.4395 | | | 3,5 | |
| S_8_ | E | hM4D(Gi)-CNO | | | Unpaired | 0.5506 | | | 0.5920 | | | | 3,5 | |  | | S_7_ | L | V3-hM4D(Gi)-CNO | Unpaired | 0.2299 | | 0.8239 | | | 3,5 | |
| S_8_ | F | Tacr3-control | | | Unpaired | 0.0523 | | | 0.9596 | | | | 5,3 | |  | | S_8_ | A | Tacr3-control | Unpaired | 0.9419 | | 0.3738 | | | 5,3 | |
| S_8_ | F | Tacr3-over | | | Unpaired | 0.0841 | | | 0.9350 | | | | 3,5 | |  | | S_8_ | A | Tacr3-over | Unpaired | 0.5362 | | 0.5739 | | | 5,3 | |
| S_8_ | F | hM4D(Gi)-PBS | | | Unpaired | 0.1183 | | | 0.9092 | | | | 3,4 | |  | | S_8_ | A | hM4D(Gi)-PBS | Unpaired | 0.2004 | | 0.8469 | | | 5,3 | |
| S_8_ | F | hM4D(Gi)-CNO | | | Unpaired | 0.4266 | | | 0.6809 | | | | 3,5 | |  | | S_8_ | A | hM4D(Gi)-CNO | Unpaired | 0.5899 | | 0.5715 | | | 5,3 | |
